# Dissecting the Regulatory Microenvironment of a Large Animal Model of Non-Hodgkin Lymphoma: Evidence of a Negative Prognostic Impact of FOXP3^+^ T Cells in Canine B Cell Lymphoma

**DOI:** 10.1371/journal.pone.0105027

**Published:** 2014-08-13

**Authors:** Dammy Pinheiro, Yu-Mei Chang, Hannah Bryant, Balazs Szladovits, Tim Dalessandri, Lucy J. Davison, Elizabeth Yallop, Emily Mills, Chiara Leo, Ana Lara, Anneliese Stell, Gerry Polton, Oliver A. Garden

**Affiliations:** 1 Department of Clinical Sciences and Services, Immune Regulation Laboratory, The Royal Veterinary College, London, United Kingdom; 2 Research Office, The Royal Veterinary College, London, United Kingdom; 3 Department of Pathology and Pathogen Biology, The Royal Veterinary College, Hatfield, Hertfordshire, United Kingdom; 4 Henry Wellcome Building, Centre for Cellular and Molecular Physiology, University of Oxford, Oxford, United Kingdom; 5 Department of Veterinary Medicine, University of Cambridge, Cambridge, United Kingdom; 6 Clinical Investigation Centre, The Royal Veterinary College, Hatfield, Hertfordshire, United Kingdom; 7 Department of Clinical Sciences and Services, Queen Mother Hospital for Animals, The Royal Veterinary College, Hatfield, Hertfordshire, United Kingdom; 8 Oncology Service, North Downs Specialist Referrals, Bletchingley, Surrey, United Kingdom; University of North Carolina at Chapel Hill, United States of America

## Abstract

The cancer microenvironment plays a pivotal role in oncogenesis, containing a number of regulatory cells that attenuate the anti-neoplastic immune response. While the negative prognostic impact of regulatory T cells (Tregs) in the context of most solid tissue tumors is well established, their role in lymphoid malignancies remains unclear. T cells expressing FOXP3 and Helios were documented in the fine needle aspirates of affected lymph nodes of dogs with spontaneous multicentric B cell lymphoma (BCL), proposed to be a model for human non-Hodgkin lymphoma. Multivariable analysis revealed that the frequency of lymph node FOXP3^+^ T cells was an independent negative prognostic factor, impacting both progression-free survival (hazard ratio 1.10; p = 0.01) and overall survival (hazard ratio 1.61; p = 0.01) when comparing dogs showing higher than the median FOXP3 expression with those showing the median value of FOXP3 expression or less. Taken together, these data suggest the existence of a population of Tregs operational in canine multicentric BCL that resembles thymic Tregs, which we speculate are co-opted by the tumor from the periphery. We suggest that canine multicentric BCL represents a robust large animal model of human diffuse large BCL, showing clinical, cytological and immunophenotypic similarities with the disease in man, allowing comparative studies of immunoregulatory mechanisms.

## Introduction

Non-Hodgkin lymphoma (NHL), a heterogeneous group of lymphoid malignancies [1], [2], is the eleventh most common cause of death from cancer in humans worldwide, estimated to be responsible for approximately 192,000 deaths in 2008 alone [3]. Despite advances in therapy in recent years, five-year survival rates are as low as 25% for the more aggressive subtypes of NHL [3]. There is thus an urgent need to develop novel, targeted therapies for this group of diseases, driven by advances in our understanding of their pathogenesis and specific prognosis. Current rodent models of lymphoma are far from predictive of the natural course of the human disease, relying either on the subcutaneous implantation of xenogeneic lymphoma cells into immune-compromised mice, or on genetic manipulations that artificially increase the likelihood of lymphoma in a monogenic fashion [4]. Key to advancing the field will be the development of natural, polygenic animal models of NHL, allowing the further interrogation of molecular and cellular pathogenesis, as well as trials of novel anti-cancer agents. The dog has gained traction in recent years as a model for a number of human diseases, including various malignancies [5]–[8]. Its spontaneous development of mesenchymal, epithelial and round cell tumors, relatively short lifespan, and cohabitation of our environment, as well as the availability of advanced diagnostic and therapeutic modalities that are similar to those available in human oncology clinics, all make this species attractive as a model for cancer research [7], [9], [10]. Furthermore, the most prevalent subtype of canine lymphoma, diffuse large (DL) BCL, mirrors the most common form of NHL [11], and various studies suggest that the molecular pathogenesis of DLBCL in the two species is fundamentally similar [12]–[15].

One of the key pathogenic determinants in oncogenesis is the cancer microenvironment [16]–[20]. Interactions with stromal and immune cells and extracellular matrix components conspire to inhibit anti-neoplastic immune responses, allowing the cancer to grow and metastasize. Central among such cellular interactions are those occurring with Tregs, of which the thymic subset is generally identified by its expression of the Forkhead box transcription factor FOXP3 in addition to the classical markers CD4 and CD25^high^ [21]. While the negative prognostic impact of FOXP3^+^ Tregs in various solid tumors of both mesenchymal and epithelial origin has been well documented [22], [23], these cells have also been associated with a favorable outcome in certain solid tumors [24], [25] and their role in the pathogenesis of lymphoid malignancies is much less clear [26]. Some papers suggest that intra-tumoral Tregs predict a negative outcome in human lymphoma [27]–[31], while others suggest that they may have a beneficial effect [32]–[40]; yet others propose that they have neither a positive nor a negative prognostic impact [41]. The situation is further complicated by the presence of more than one subset of Treg – including both thymic and peripheral IL-10-secreting (Tr1) phenotypes – and interactions with other cells with suppressive properties, including myeloid-derived suppressor cells, natural killer T (NKT) cells and tolerogenic dendritic cells [42]–[45].

While a number of recent studies have added weight to the suggestion that lymphoma in the dog closely resembles human NHL [12]–[15], very little is known about the role of Tregs in canine BCL. We and others have recently characterised FOXP3^+^ Tregs in the dog [46], demonstrating that they have similar properties to those of human Tregs; we were thus optimally poised to interrogate their role in various canine diseases, including lymphoma. The aim of the present study was therefore to document the presence of FOXP3^+^ Tregs in the resident T cell population of affected lymph nodes of dogs with B cell lymphoma (BCL) and to test the hypothesis that intra-tumoral CD4^+^FOXP3^+^ Tregs have a negative impact on treatment outcome, making comparisons with dogs with T cell lymphoma (TCL), non-neoplastic causes of lymphadenomegaly, and metastatic mast cell tumors (MCTs). Our goal was to advance our understanding of the pathogenesis of canine BCL, thus further validating this spontaneous, naturally occurring disease as a large animal model of NHL in which to study the role of immunoregulatory pathways.

## Materials and Methods

### Case accession

Dogs were recruited to this study from four different veterinary practices, three located in the UK and one in Sweden. Complete clinical histories were obtained from every dog. Diagnostic investigations appropriate to the presenting complaints were undertaken in all dogs, including cytological and, or histopathological review of enlarged lymph nodes by a board-certified veterinary pathologist. Standard clinical staging was undertaken by means of thoracic radiographs or computed tomography; abdominal ultrasound examination; fine needle aspirates (FNAs) of the liver and spleen when sonographic lesions of these organs were identified; and bone marrow aspirates when prompted by peripheral cytopenias. Stages IV and V were thus assigned with the caveat that aspirates of the liver, spleen and bone marrow were not undertaken in every dog. Study samples comprised blood collected by venepuncture of a peripheral vein into EDTA and serum gel tubes, and three to four fine needle aspirates (FNAs) of a representative, enlarged lymph node deposited into neat fetal bovine serum (PAA Laboratories Ltd, Yeovil, UK) for flow cytometry. Staining of cells for flow cytometry was performed within 48 hours of sample collection, followed by acquisition of the fixed cells within a further 72 hours. (Pilot studies had established that the immunophenotype of samples remained stable up to 48 hours after sample collection.) Serum was harvested within two hours of sample collection and was stored at a temperature of −20°C until analysis.

Twenty-two of the dogs recruited were diagnosed with BCL, whilst the remaining cases were recruited to three different control groups. The first comprised 14 dogs with TCL; the second comprised 14 dogs with reactive hyperplasia (RH) associated with cutaneous inflammation/dermatitis, immune-mediated disease, distant neoplasia (i.e. non-draining lymph nodes were sampled) or systemic infection; while the third comprised six dogs with metastatic MCTs, the samples in these cases having been collected from draining lymph nodes. Flow cytometric analyses of peripheral blood were undertaken in these dogs and a group of 25 healthy control (HC) dogs, which also contributed serum samples. (C-reactive protein and cytokine assays, reported elsewhere, were undertaken on all of the serum samples.) For ethical and legal reasons, lymph node samples could not be obtained from HC dogs. All patient samples were collected by licensed veterinarians in routine diagnostic fashion following written informed consent and all study protocols were approved by the Royal Veterinary College Ethics and Welfare Committee (Permit Number: URN-20101005). None of the dogs suffered any adverse consequences of sample procurement.

### Cytomorphological classification

When available, slides of diagnostic specimens were retrieved from the receiving diagnostic laboratories for review by a board-certified clinical pathologist involved in the study (BS) for cytomorphological classification. Cytological features that were recorded in the study are presented in Table S1. Based on these observations and the immunophenotype of the lymphoma, the most likely subtype was deduced – for example, DLBCL – though histopathological review would have been required to confirm such tissue diagnoses.

### Flow cytometry

A panel of canine-specific or cross-reactive fluorochrome-conjugated monoclonal antibodies (mAbs) against both extra- and intra-cellular antigens was applied (Table 1). The manufacturer's protocol for FOXP3 staining was applied. Briefly, lymphocytes were washed and stained with mAbs for 20 minutes on ice. Lymphocytes were then washed and incubated overnight in a 1∶4 (concentrate:diluent) fixation/permeabilization solution. Following incubation, the lymphocytes were washed twice and then stained with mAbs against intracellular antigens for 30 minutes on ice. All incubations were carried out in the dark. Upon completion, lymphocytes were washed twice and re-suspended in 200 µl phosphate buffered saline (PBS) solution. Isotype control antibodies were used to define gates in all experiments. Three-colour staining was used throughout the study, T cells identified in a panel comprising mAbs against CD5 and FOXP3 with CD4, CD8 or Helios. Data were acquired using a FACS Canto II flow cytometer (BD Biosciences) and were analyzed using Flowjo (Tree Star Inc) software.

**Table 1:** Staining antibodies, isotype controls and fluorochromes.

Antigen	Clone	Isotype	Fluorochrome	Supplier
CD5	YKIX322.3	Rat IgG2a	FITC/PE	AbD Serotec, UK
CD4	YKIX302.9	Rat IgG2a	FITC/PE	AbD Serotec, UK
CD8	YCATE55.9	Rat IgG1	FITC/PE	AbD Serotec, UK
CD79b	AT107-2	Rat IgG1	FITC	AbD Serotec, UK
CD21	CA2.1D6	Mouse IgG1	PE	AbD Serotec, UK
CD34	1H6	Mouse IgG1	PE	AbD Serotec, UK
FOXP3	FJK-16s	Rat IgG2a	APC	eBioscience, UK
Helios	22F6	Armenian hamster IgG	PE	Biolegend, UK

Abbreviations: FITC, fluorescein isothiocyanate; PE, phycoerythrin; APC, allophycocyanin.

### Immunophenotypic determination

**Figure 1:**
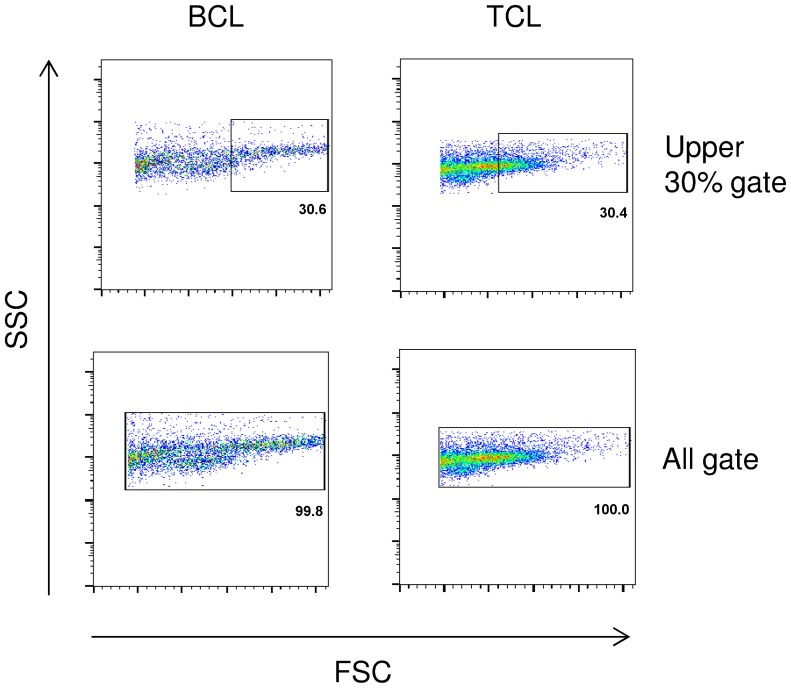
Gating strategies used to interrogate the neoplastic and non-neoplastic cell populations. Following exclusion of dead cells and debris, two different gating strategies were employed to evaluate the cells isolated from lymph node aspirates. For the purposes of generating an immunophenotyping profile, an upper 30% gate was applied to identify the population of largest events, enriched in neoplastic cells. The immunophenotype was then determined using a 60% expression threshold within this population. For the purposes of evaluating frequencies of T cells within fine needle aspirates of B cell lymphoma (BCL), reactive hyperplasia (RH) and mast cell tumour (MCT) cases, an ‘All’ gate was applied and then the populations subsequently delineated by means of CD5 expression. Non-neoplastic cells were identified as CD5^+^ and neoplastic populations CD5^−^ in the context of BCL; TCL cases were excluded from such comparisons on account of their CD5 expression by both neoplastic and non-neoplastic cells.

A threshold of 60% marker expression was used to classify patient samples as being of either a B or T cell phenotype, adopting a previously validated diagnostic protocol [47]. This threshold was applied to the 30% of largest events following removal of dead cells and debris by appropriate gating, as determined by forward and side scatter characteristics (Figure 1). Where at least 60% of cells expressed CD21 or CD79b, a classification of BCL was made. Conversely, when at least 60% of cells expressed CD5, CD4 or CD8 a classification of TCL was made. To further interrogate the composition of cell populations in FNAs, an ‘All’ gate was applied, which again excluded dead events. Non-neoplastic and neoplastic populations of cells within the BCL FNAs were then evaluated on the basis of CD5 expression, which was limited to the resident (non-neoplastic) T cells in the majority of such cases. An equivalent strategy was applied to both the RH and MCT groups; however, TCL samples were excluded from this analysis owing to CD5 expression by both neoplastic and non-neoplastic cells, which precluded distinction of the two populations within the aspirated cells. In all cases, the expression of CD34 in peripheral blood was negligible, ruling out leukemia.

### Clinical definitions and statistical analysis

The following outcome metrics were derived from patient records and communications with the clinicians involved in this study: time to remission (TTR), progression-free survival (PFS) and overall survival (OS). These parameters were determined following assessment of the response to therapy using Veterinary Cooperative Oncology Group-approved definitions of complete response (CR), partial response (PR), stable disease (SD) and progressive disease (PD) applied to peripheral lymph nodes measured by the attending clinicians [48].

Comparisons between two groups of continuous data were undertaken using Mann-Whitney U tests and for three or more groups using Kruskal-Wallis tests. Summary data are presented in the text in the following format: median, range [lowest to highest value], number of cases or experimental repeats (n); p value. Summary data are presented in the form of box plots, in which the boxes show the respective 25^th^ and 75^th^ percentiles, the horizontal lines median values, and the whiskers, the lowest and highest data points still within 1.5 times the interquartile range of the respective lower and upper quartiles. Dots represent the data from individual dogs. Red symbols correspond to data from dogs ‘pre-treated’ with corticosteroids or any chemotherapeutic drug within three weeks of sampling. In the case of RH cases, square symbols represent data from dogs with *dermatitis*; diamonds represent data from dogs with *distant neoplasia*; triangles represent data from dogs with *immune-mediated disease*; and inverted triangles represent data from dogs with *systemic infection*.

Survival analysis of the dogs with lymphoma was performed using Cox's proportional hazards regression and presented in the form of Kaplan-Meier curves. Continuous variables were categorised to produce the Kaplan-Meier curves using the median value as a threshold. Both univariable and multivariable analyses were undertaken. Criteria for inclusion of variables into the multivariable model included significance at the level of univariable analysis, lack of overlap of the respective survival curves, and constancy of the risk (hazard) with time. Only the BCL cases were analyzed as a separate group for the prognostic impact of phenotypic variables; there were too few TCL cases to make survival analysis of phenotypic variables of this group meaningful. Dogs that failed to go into complete remission were censored for TTR analysis; those that did not show progression, including death, at the last known follow-up time point were censored for PFS analysis; and those that were still alive at the last known follow-up time point were censored for OS analysis. Rescue therapy was not a criterion for censorship in the OS analysis.

## Results

### Clinical and immunophenotypic details of cases and controls

A total of 22 BCL, 14 TCL, 14 RH, six MCT and 25 HC dogs were recruited into the study. A summary of the signalment and clinical details of the BCL, TCL, RH, MCT and HC cases is presented in Table 2; further details are presented in Tables S2 to S5.

**Table 2:** Clinical, cytological and treatment details of dogs in the study.

		B cell lymphoma	T cell lymphoma	Reactive hyperplasia	Mast cell tumor	Healthy control dogs
**Age (months)^1^**	Median (range)	113 (33–171)	93 (58–163)	108 (15–156)	138 (84–176)	55 (19–324)
**Sex**	Me	6	4	1	0	2
	Mn	7	3	6	0	11
	Fe	4	1	1	1	2
	Fn	5	6	6	5	10
**Breed**		Labrador retriever (n = 2); Afghan hound, Australian silky, Bernese mountain dog, Border collie, bullmastiff, cocker spaniel, Doberman, English springer spaniel, flat-coated retriever, German shepherd dog, golden retriever, Jack Russell terrier, Shar pei, Shetland sheepdog, shih tzu, spaniel cross, Staffordshire bull terrier, Tibetan terrier, undefined cross breed, West Highland white terrier (all n = 1)	Boxer (n = 4); Labrador retriever (n = 3); cocker spaniel (n = 2); Border collie, Dogue de Bordeaux, English springer spaniel, golden retriever, Shetland sheepdog (all n = 1)	Fox terrier (n = 2); Labrador retriever (n = 2); Border collie, cocker spaniel, French bulldog, German shepherd dog, Irish terrier, Jack Russell terrier, Labrador cross, Patterdale terrier, Rottweiler, undefined cross breed (all n = 1)	Golden retriever (n = 2); Labrador retriever (n = 2); Rottweiler, undefined cross breed (all n = 1)	Rhodesian ridgeback (n = 3); Border terrier (n = 2); Jack Russell terrier (n = 2); Labrador retriever (n = 2); whippet (n = 2); greyhound, lurcher, standard poodle, boxer cross, Dogue de Bordeaux, Hungarian vizsla, English springer spaniel, Japanese akita, Labrador/English springer spaniel cross, Staffordshire bull terrier (all n = 1); undefined cross breed (n = 4)
**Body condition^2^**	Under	4	1	2	0	0
	Optimal	13	9	10	4	24
	Over	5	4	2	2	1
**WHO stage**	II	0	1	N/A	N/A	N/A
	III	9	11	N/A	N/A	N/A
	IV	7	2	N/A	N/A	N/A
	V	6	0	N/A	N/A	N/A
**Sub-stage**	a	11	6	N/A	N/A	N/A
	b	11	8	N/A	N/A	N/A
**Hyper-calcemic?** ^3^	No	21	9	N/A	N/A	N/A
	Yes	1	4	N/A	N/A	N/A
**Cytological appearance**		DLBCL: n = 11; DLBCL or Burkitt-type: n = 2; Undefined: n = 1; Slides not available for review: n = 8	PTCL: n = 8; Small cell: n = 1; T zone: n = 1; Undefined: n = 1; Slides not available for review: n = 3	Reactive lymphoid hyperplasia confirmed in every case	Metastatic mast cell tumor confirmed in every case	N/A
**Chemotherapy protocol^4^**	CHOP	14	8	Various drugs administered, including antimicrobials, NSAIDs and prednisolone	Various drugs administered, including vinblastine, prednisolone, lomustine, masitinib and toceranib	N/A
	COP	3	1	N/A	N/A	N/A
	Other	3	4	N/A	N/A	N/A
	Pred	1	0	N/A	N/A	N/A
	None	1	1	N/A	N/A	N/A

Abbreviations: Me, entire male; Mn, neutered male; Fe, entire female; Fn, neutered female; WHO, World Health Organization; N/A, not applicable; DLBCL, diffuse large B cell lymphoma; PTCL, peripheral T cell lymphoma; CHOP, cyclophosphamide, doxorubicin, vincristine, prednisolone protocol; COP, cyclophosphamide, vincristine, prednisolone protocol; Pred, prednisolone alone; NSAIDS, non-steroidal anti-inflammatory drugs.

Notes: ^1^: Age when presenting signs were first observed. ^2^: Body condition score was assessed by different clinicians on either a 5 or 9 point scale: ‘under-conditioned’ was defined by a score of 1–2/5 or 1–3/9; ‘optimal’ was defined by a score of 2.5–3/5 or 4–5/9; while ‘over-conditioned’ was defined by a score of 3.5–5/5 or 6–9/9. ^3^: Serum or plasma calcium concentration was not measured in one of the T cell lymphoma cases prior to initiation of therapy. ^4^: The initial treatment protocol is listed. ‘Other’ treatments included cytarabine, L-asparaginase and lomustine (B cell lymphoma) and L-asparaginase, lomustine, prednisolone, masitinib and chlorambucil (T cell lymphoma).

Comparing the BCL, TCL, RH and MCT dogs, there was no significant difference in age (p = 0.51), sex (p = 0.14), neutering status (p = 0.27), bodyweight (p = 0.09) or body condition (p = 0.86) between the groups. The BCL dogs tended to be of higher stage (III 41%, IV 32%, V 27%) than the TCL (II 7%, III 79%, IV 14%; p = 0.02), but there was no difference in sub-stage between the groups (p = 1.00). In common with the prevailing literature, TCL dogs were more commonly hypercalcemic (4/13; 31%) than BCL (1/22, 4.5%; p = 0.05). Among the lymphoma dogs, the cytomorphological characteristics when considered alongside immunophenotype were most commonly consistent with DLBCL (11/22; 50%) and peripheral TCL (8/14, 57%; Table 2, Figure S1). The most common chemotherapy protocol in both BCL and TCL dogs was CHOP, comprising cyclophosphamide, doxorubicin, vincristine and prednisolone (Tables 1, S2 and S3).

Treatment of the BCL and TCL dogs with a corticosteroid or any cytotoxic chemotherapeutic drugs within three weeks of sampling was considered a confounding factor, since such drugs may theoretically alter the frequency of peripheral Tregs. Of the BCL cases, 9/21 (43%) dogs had been ‘pre-treated’ in this way, compared to 5/13 (38%) of the TCL cases. (In each respective BCL and TCL group, one dog received no treatment at all.) Phenotypic characteristics of the pre-treated dogs were no different from those that had not received such drugs prior to sampling (BCL, p>0.05; TCL, p>0.05) and pre-treatment had no impact on outcome variables (TTR: BCL, p = 0.58; TCL, p = 0.46; PFS: BCL, p = 0.30; TCL, p = 0.11; OS: BCL, p = 0.28; TCL, p = 0.91). The pre-treated and treatment-naïve groups were therefore considered as a single group in respective BCL and TCL cases. A complete response to chemotherapy was achieved in 17/21 (81%) of the BCL cases and in 8/13 (62%) of the TCL cases. Thus, four of the BCL and five of the TCL dogs were censored from TTR analysis. One dog in each lymphoma group was censored from PFS analysis, while five BCL and two TCL dogs were censored from OS analysis. Comparing the BCL with TCL groups, there was no difference in TTR (BCL: median 20 days *versus* TCL: median 27 days; p = 0.09), PFS (BCL: median 117 days *versus* TCL: median 63 days; p = 0.47) or OS (BCL: median 294 days *versus* TCL: median 151 days; p = 0.21). In the case of the BCL group, older dogs had shorter PFS (median 74 days) than younger dogs (median 174 days; p = 0.006) and dogs treated with CHOP had longer PFS (median 175 days) than those treated with the other protocols (median 45 days; p = 0.01) (Figure S2). Rescue therapy, which was unstandardized, was administered to 10 of the BCL dogs and nine of the TCL dogs (Tables S2 and S3). In the case of both the BCL and TCL groups, dogs receiving rescue therapy had longer OS than those not receiving it (BCL: median OS 322 days *versus* 174 days respectively, p = 0.049; TCL: median OS 230 days *versus* 9.5 days respectively, p = 0.008; combined BCL+TCL: 294 days *versus* 102 days respectively, p = 0.013). There was no association with prognosis in either the BCL or the TCL groups for sex, neutering status, body weight, body condition, stage, substage or presence of hypercalcemia (in all cases p>0.05).

### Neoplastic effacement of normal lymphoid tissue impacts FOXP3, Helios and CD8 expression, but the ratio of cytotoxic to regulatory T cells is not decreased in canine B cell lymphoma

**Figure 2:**
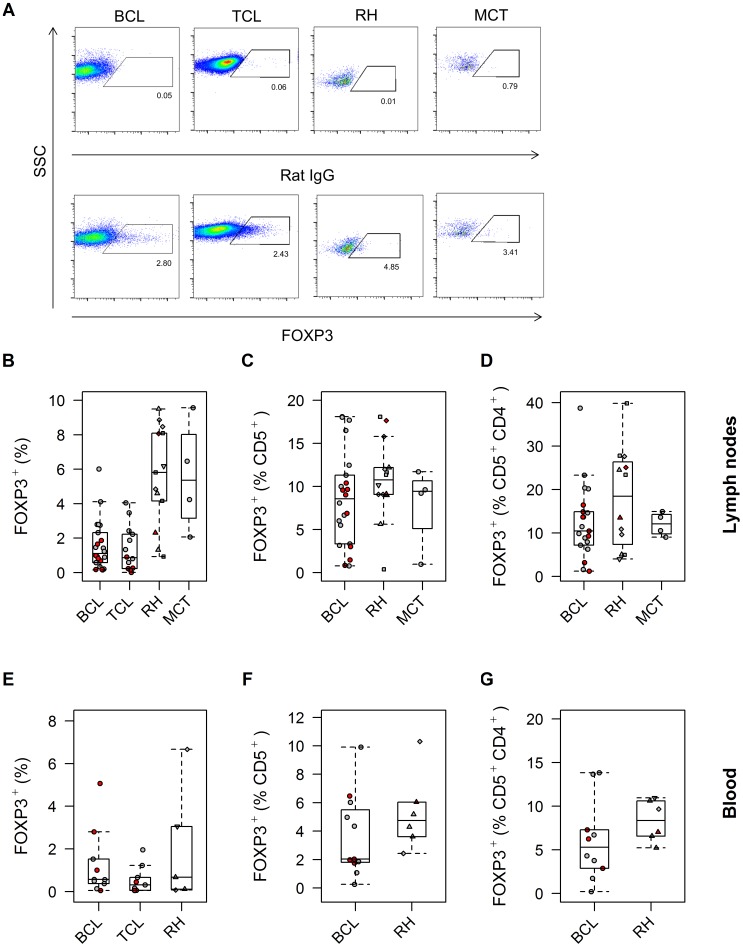
FOXP3^+^ Treg frequency is no higher in canine B cell lymphoma than control samples. The frequency of FOXP3^+^ cells in fine needle aspirates harvested from the lymph nodes of dogs with B cell lymphoma (BCL), T cell lymphoma (TCL), reactive hyperplasia (RH) and mast cell tumors (MCT) is shown, expressed as a proportion of all cells (A, B, showing the effect of neoplastic effacement of normal lymphoid tissue; p = 0.00005), gated CD5^+^ cells (C, showing the resident T cells; p = 0.41), or sequentially gated CD5^+^ and then CD4^+^ T cells (D, showing specifically the resident CD4^+^ T cells; p = 0.74). Representative dot plots are shown for each of the groups, followed by box-and-whisker plots summarising the data. In each of the dot plots, the respective isotype control stain is shown above the specific stain. In this and subsequent figures, red symbols correspond to data from dogs ‘pre-treated’ with corticosteroids or any chemotherapeutic drug within three weeks of sampling. In the case of RH cases, square symbols (□) represent data from dogs with dermatitis; diamonds (◊) represent data from dogs with distant neoplasia; triangles (Δ) represent data from dogs with immune-mediated disease; and inverted triangles (▾) represent data from dogs with systemic infection. The expression of FOXP3 by PBMCs (E; p = 0.21) or by the CD5^+^ (F; p = 0.53) or specific CD5^+^CD4^+^ (G; p = 0.15) T cells within PBMCs similarly showed no differences between groups.

The median frequency of FOXP3^+^ cells within the whole population of cells harvested by fine needle aspiration, identified by the ‘All’ gate, was significantly lower in BCL (1.1% [0.16%–6.01%]; n = 22) and TCL (0.86% [0.01%–5.15%]; n = 14) cases than in both RH (6.06% [0.93%–9.50%]; n = 13) and MCT (5.36% [2.52%–9.57%]; n = 4) cases (p = 0.00005; Figure 2A,B). This was attributed to effacement and thus ‘dilution’ of normal lymphoid tissue by tumor cells, prompting further examination of FOXP3 expression specifically within the T cell population of BCL, RH and MCT cases. A similarly defined sub-population of non-neoplastic cells could not be delineated in TCLs owing to CD5 expression by the neoplastic cells themselves, precluding such analysis in the TCL cases.

A variable pattern of FOXP3 expression was observed in BCL lymph node aspirates, but there was no overall difference between groups when FOXP3 was examined in either the CD5^+^ population (BCL: 8.56% [0.77%–18.1%], n = 22; RH: 10.8% [0.37%–18.1%], n = 14; MCT: 9.43% [0.96%–11.7%], n = 4; p = 0.41) or the specific CD4^+^CD5^+^ T cells (BCL: 6.21% [0.57%–17.1%], n = 22; RH: 6.79% [0.38%–13.4%], n = 14; MCT: 9.51% [5.64%–10.6%], n = 4; p = 0.74) within the lymph nodes (Figure 2C,D). Furthermore, there was no overall difference in expression of FOXP3 by PBMCs between groups (BCL: 0.57% [0.05%–5.07%], n = 10; TCL: 0.31% [0.05%–1.96%], n = 9; RH: 0.67% [0.07%–6.67%], n = 5; MCT: 12.04%, n = 1; p = 0.21) (Figure 2E). A similar lack of difference in FOXP3 expression between groups was also apparent when its expression within the CD5^+^ (BCL: 2.03% [0.25%–9.91%], n = 11; TCL: 4.31% [0.18%–6.48%], n = 8; RH: 4.74% [2.43%–10.30%], n = 6; MCT: 5.9%, n = 1; p = 0.53) or specific CD4^+^CD5^+^ T cell fractions of PBMCs was examined (BCL: 5.29% [0.21%–13.85%], n = 10; TCL: 5.41% [0.258%–16.02%], n = 9; RH: 8.36% [5.23%–10.96%], n = 6; MCT: 16.13%, n = 1; p = 0.15) (Figure 2F,G).

**Figure 3:**
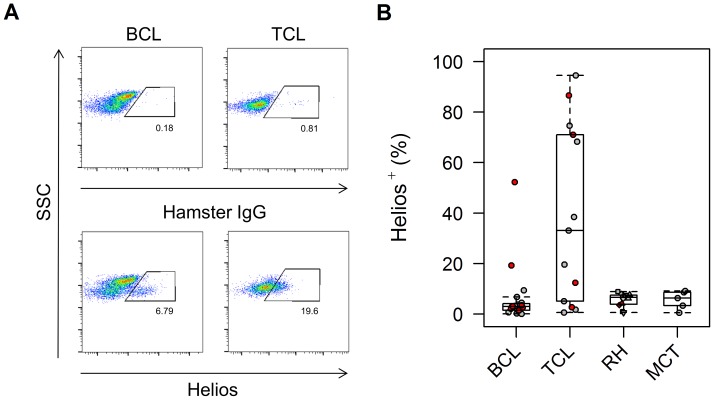
Canine T cell lymphoma is associated with high-frequency Helios expression. Helios expression by cells harvested by fine needle aspiration from the lymph nodes of dogs with B cell lymphoma (BCL), T cell lymphoma (TCL), reactive hyperplasia (RH) and mast cell tumors (MCT) was examined as a marker for thymic Tregs, but the unexpectedly high frequency of Helios^+^ events in a few B cell and several T cell lymphoma cases suggested neoplastic expression of this marker. Median frequency of Helios expression was significantly different between the groups (p = 0.00003). Representative dot plots for BCL and TCL cases, with their respective isotype control stains, is shown (A), followed by box-and-whisker plots summarising the data (B). (For key to symbols, refer to Figure 2).

The median frequency of expression of Helios within the whole population of aspirated cells was markedly higher in TCL cases (33.1% [0.6%–94.0%]; n = 13) than in the other three groups of dogs (BCL: 3.0% [0.1%–52.3%], n = 22; RH: 6.65% [0.60%–8.90%], n = 12; MCT: 6.38% [0.50%–9.13%], n = 5; p = 0.00003), compatible with neoplastic T cell Helios expression rather than localization of Helios to the thymic Tregs alone (Figure 3A,B).

Expression of Helios was therefore of limited use in the identification of thymic Tregs in TCL.

**Figure 4:**
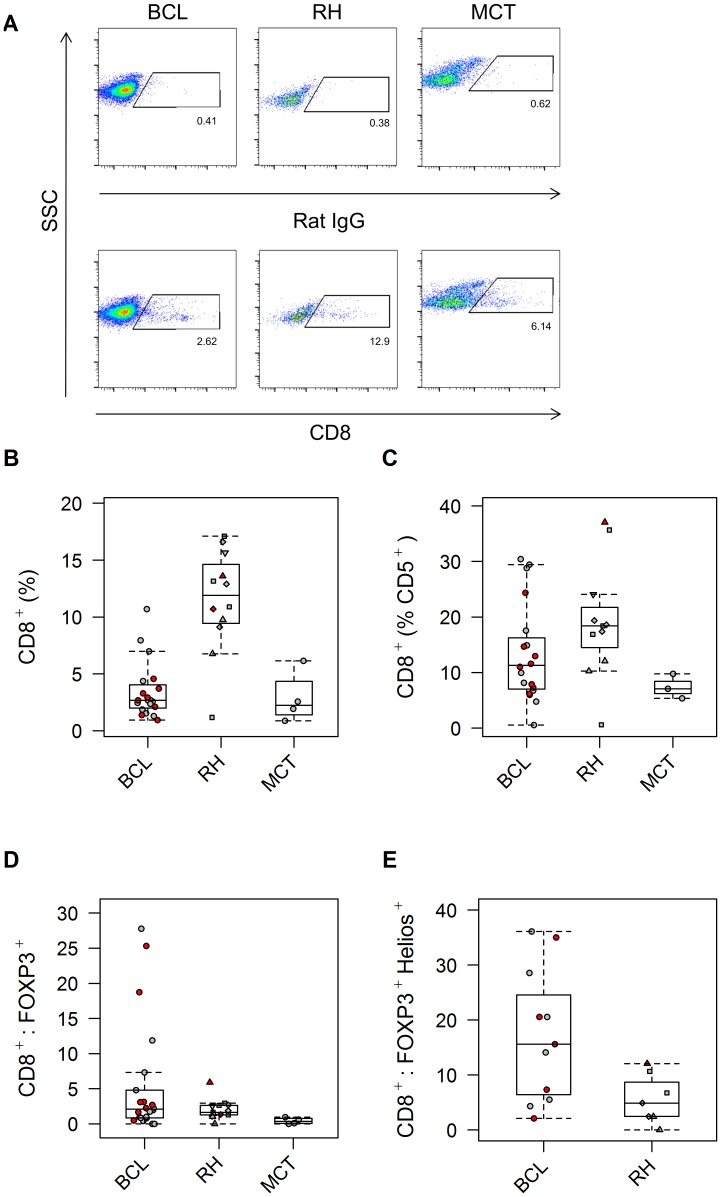
Canine B cell lymphoma shows no deficit of cytotoxic T cells relative to thymic Tregs. When expressed as a proportion of all cells aspirated from the lymph node, the frequency of CD8^+^ T cells (A,B) was decreased in BCL (and MCT), again attributed to neoplastic effacement (p = 0.0005). The frequency of CD8^+^ cells within the CD5^+^ T cell population also showed differences between groups (C; p = 0.04). The ratio of CD8^+^:FOXP3^+^ cells (D) was not significantly different between groups (p = 0.06), though dogs with BCL showed the six highest ratios of the cohort, and a trend for higher values in the BCL group was observed. Indeed, the ratio of CD8^+^:FOXP3^+^Helios^+^ cells (E) was higher in the BCL than RH cases (p = 0.04); only two data points for the MCT (3.48; 3.95) were available for this variable and were not therefore represented. Representative dot plots for all the groups are shown alongside their respective isotype controls, followed by box-and-whisker plots summarising the data. (For key to symbols, refer to Figure 2).

The median frequency of CD8^+^ cells within the whole population of harvested cells was significantly lower in the BCL cases (2.68% [0.89%–10.7%]; n = 20) than in RH cases (11.1% [1.17%–17.1%], n = 20; p = 0.0005), again attributed to effacement of the normal lymphoid population by neoplastic cells (Figure 4A,B). In the case of this marker, median expression was also low in the MCT cases (2.24% [0.82%–6.9%]; n = 4). The median expression of CD8 specifically by T cells was also lower in the neoplastic groups than in the RH group (BCL: 11.3% [0.57%–30.4%], n = 20; MCT: 7.06% [5.35%–9.76%], n = 3; RH: 18.4% [0.6%–37.1%], n = 11; p = 0.04) (Figure 4C). Rationalizing that the balance of cytotoxic to regulatory T cells may be perturbed in BCL, we then examined the ratio of CD8^+^ to FOXP3^+^ T cells within the whole population of harvested cells. Rather than being lower in BCL cases, the median CD8^+^:FOXP3^+^ ratio showed a trend towards being higher in this group of dogs when compared to RH (or MCT) cases (BCL: 2.09 [0–27.7], n = 22; RH: 1.63 [0–5.89], n = 13; MCT: 0.35 [0–1.07]; n = 4; p = 0.06), which became significant when thymic Tregs were specifically examined by means of co-expression of FOXP3 and Helios within the overall population (BCL: 15.6 [2.11–36.1], n = 11; RH: 4.26 [0–12.0], n = 13; p = 0.04) (Figure 4D,E).

### FOXP3, Helios and MHC class II expression all have prognostic significance in canine B cell lymphoma

**Figure 5:**
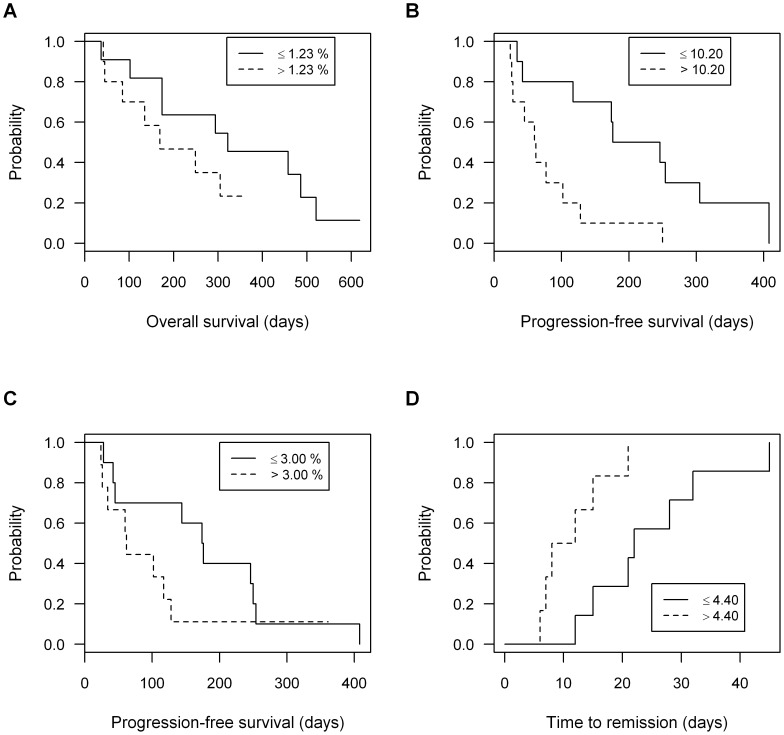
Expression of T cell FOXP3 and Helios, and tumor cell MHC class II, all impact prognosis in dogs with B cell lymphoma. The frequency of FOXP3^+^ cells within the ‘All’ gate (A), or within a cascaded All→CD5^+^→CD4^+^ gate (B), influenced survival times. Overall survival times were shorter in those dogs with frequencies of FOXP3^+^ T cells higher than the median value (A: p = 0.02); a similar pattern in progression-free survival (PFS) was observed (B: p = 0.01). The frequency of Helios^+^ cells within the ‘All’ gate also influenced PFS (C: p = 0.01). The intensity of MHC class II expression by the lymphoma cells, expressed as the ratio of geometric mean fluorescence intensity of MHC II^+^:MHC II^−^ cells within the CD5^−^ subset of the ‘All’ gate, was also of prognostic significance: time to remission was longer in those cases with less MHC class II expression per cell (D: p = 0.02).

The impact of FOXP3, Helios and MHC class II expression on BCL prognosis was then interrogated using both univariable and multivariable analysis. FOXP3 expression was a negative prognostic factor in the context of OS and PFS. Patients with total FOXP3 expression less than or equal to the median value (1.23%) survived longer than those with expression above the median value (≤1.23%, median OS = 322 days, >1.23%, median OS = 169 days; median ratio 0.52; p = 0.018) (Figure 5A). A similar phenomenon was observed for PFS: patients with lower FOXP3 expression in the CD5^+^CD4^+^ population had a longer PFS than those with higher FOXP3 expression (≤10.2%, median PFS = 211 days, >10.2%, median PFS = 61 days; median ratio 0.29; p = 0.012) (Figure 5B). The proportional expression of Helios was also a negative prognostic factor, as patients with expression lower than the median value had longer PFS (≤3.00%, median PFS = 175 days, >3.00%, median PFS = 62 days; median ratio 0.35; p = 0.014) (Figure 5C). The intensity of MHC class II expression by the lymphoma cells, expressed as the ratio of geometric mean fluorescence intensity of MHC II^+^:MHC II^−^ cells within the CD5^−^ subset of the ‘All’ gate, was also of prognostic significance (Figure 5D): TTR was longer in those cases with expression ratios less than or equal to the median value (≤4.40, median 22 days, >4.40, median 10 days; p = 0.017).

Multivariable analysis yielded two independent factors impacting PFS, age and frequency of FOXP3 expression by lymph node CD5^+^CD4^+^ T cells, and two independent factors impacting OS, the administration of rescue therapy and the frequency of FOXP3 expression within the total harvested population (Table 3). In the case of OS analysis, a similar proportion of dogs in the FOXP3^low^ (≤1.23%: 6/11) and FOXP3^high^ (>1.23%: 4/10) group had received rescue therapy (p = 0.67). Univariable analysis identified only a single factor impacting TTR (MHC class II expression), obviating the need for multivariable analysis for this outcome variable.

**Table 3:** FOXP3, MHC class II, age at presentation and rescue therapy are independent prognostic factors in canine B cell lymphoma.

Outcome metric	Variable	Median ratio	Hazard ratio (95% CI)	p value
TTR	MHC class II	0.45	2.44 (1.36–4.35)	0.003
PFS	Age	0.43	1.02 (1.01–1.04)	0.007
	FOXP3^+^ (% CD5^+^CD4^+^)	0.29	1.10 (1.02–1.18)	0.01
OS	Rescue therapy	0.54	0.29 (0.09–0.90)	0.03
	FOXP3^+^ (%)	0.52	1.61 (1.11–2.32)	0.01

Abbreviations: TTR, time to remission; PFS, progression-free survival; OS, overall survival; CI, confidence interval; FOXP3^+^ (% CD5^+^CD4^+^), frequency of FOXP3^+^ cells within the sequential CD5^+^ and CD4^+^ gates; FOXP3^+^ (%), frequency of FOXP3^+^ cells within the total harvested population; median ratio =  ratio of median remission or survival time (TTR/PFS/OS) of the sub-group of dogs with a value of the variable greater than the median of the group to that of the sub-group of dogs with a value of the variable less than or equal to the median of the group.

## Discussion

To the authors' knowledge, this study represents the first attempt to define the prognostic impact of Tregs in a well-characterized cohort of canine cases of multicentric BCL. Despite the relatively small size of the cohort and heterogeneity of treatment protocols, the expression of FOXP3 emerged as an independent prognostic factor, alongside the expression of MHC class II, age at presentation and the administration of rescue therapy. This observation raises the intriguing possibility that the dog, in common with other species, harbors thymic Tregs within the neoplastic microenvironment of BCL, which are likely to interact with the neoplastic B cells, stromal cells, and other regulatory populations such as tolerogenic dendritic cells, tumor-associated macrophages, myeloid-derived suppressor cells and γδ T cells.

Control dogs included those with TCL, non-neoplastic lymphadenomegaly (RH) and metastatic MCTs, the latter as an example of an alternative round cell tumor. In common with previous literature, boxers appeared to be over-represented in the TCL group (4/14, 29%) [49]–[52] and dogs with TCL were more commonly hypercalcemic than those with BCL [53], [54]. Cytological reviews were possible in 14 of 22 BCL and 11 of 14 TCL cases, suggesting a predominance of DLBCL and peripheral TCL. This observation accords with both previous veterinary literature, in which these cytological classifications were the most commonly recognized among multicentric BCL and TCL cases [51], [55], [56], and reviews of human NHL [2], [57]. The similar cytological appearance of the most common types of canine and human BCL adds weight to the notion that the canine disease could model NHL [11]. However, although FNAs are still the cornerstone of lymphoma diagnosis in canine patients and serve an important triaging role in people with lymphadenomegaly by virtue of their ease of procurement [58]–[60], solid tissue biopsies are considered the gold standard for diagnosis of human patients, allowing a detailed tissue architectural classification of lymphoma subtypes [1], [2]. Recent years have seen an increasing recognition of the diagnostic and prognostic value of histopathological subtypes of lymphoma in dogs [56], [61], [62], which generally show remarkable similarity to those of human patients [51], [55], [63].

Most of the dogs in this study were treated with a CHOP protocol, considered to be the standard of care in the treatment of canine lymphoma [64], [65]; anti-canine CD20 mAbs similar to rituximab are not currently available, precluding R-CHOP. A proportion of the dogs had received at least one of the drugs on at least one occasion within three weeks of sampling for this study, prompting concern that such ‘pre-treatment’ might influence their immune cell profile. In particular, we were concerned that prior use of corticosteroids or cyclophosphamide would alter Treg frequencies, given the known impact of these drugs on peripheral T cells in rodents [66]–[68], humans [69]–[71] and dogs [72]. However, neither the cellular phenotype nor the prognosis of the pre-treated dogs was significantly different from that of the treatment-naïve patients, presumably because such drugs were administered on a limited number of occasions prior to sampling and would thus have had only minimal opportunity to impact the immune system. Recent studies of human patients receiving short-term glucocorticoid therapy have similarly shown no change in the frequency of peripheral Tregs [73], [74]. In view of the lack of impact of pre-treatment on these variables, all dogs of the respective B and T cell groups were considered together in our final analysis.

The impact of a number of clinical and immunophenotypic variables on TTR, PFS and OS of dogs with multicentric lymphoma was examined in the current study, including B or T cell phenotype, age, sex, neutering status, bodyweight, body condition, stage, substage, presence of hypercalcemia, treatment protocol (CHOP *versus* ‘Other’) and the administration of rescue therapy. Other than the predictable impact of rescue therapy on OS, multivariable analysis revealed that age was the only factor with an independent impact on prognosis, younger dogs enjoying longer PFS. This has been a finding in other canine studies [62] and also reflects the situation in human NHL, for which the prognosis is worse in elderly patients [75], [76]. The pathophysiological basis of this observation remains unclear, but is thought to reflect the generally poorer cellular repair mechanisms, progressive immunosenescence, propensity for chemoresistant molecular subtypes of DLBCL, and increased spectrum of comorbidities that occur with advancing age [77].

Prognostic factors in other canine studies of multicentric lymphoma have included sex [78], [79], B or T immunophenotype [53], [80]–[82], stage [81], [83]–[85], substage [79], [83], serum calcium concentration [86], packed cell volume [87], monocyte concentration [88], prior administration of corticosteroids [89], [90], treatment protocol [62], [91], coagulation profile [92], presence of minimal residual disease after treatment [93], and cytomorphological and histopathological characteristics [56], [61], [62], [81]. However, in common with our own observations, different studies have yielded different conclusions and have not all been able to demonstrate a prognostic impact of every one of the examined variables: factors such as the number and clinical heterogeneity of recruited patients and the nature of the specific end points examined influence the results of such outcome analyses [94]. More recent studies have stratified canine BCL patients by gene profiling, demonstrating that in common with human DLBCL two subtypes with different survival times – the germinal center B cell-like and the activated B cell-like – can be distinguished, the latter associated with a poorer prognosis [14], [15]. While the individual genes were different between the species, the general pathways characterizing these subtypes were strikingly similar [14].

We used a cross-reactive mAb against murine Foxp3, validated for use in dogs [46], [95], to identify Tregs in this study. While a large number of markers for Tregs have been proposed, FOXP3 remains the single most widely applied phenotypic credential of regulatory activity in cancer and other studies [96]–[98], despite being transiently expressed in activated human and canine conventional T cells lacking regulatory function [46], [99]. Comparisons of respective FOXP3^+^ and CD8^+^ cells between the study groups yielded the initially surprising observation that dogs with lymphoma had lower frequencies of these cells than those with RH and MCTs. While FNAs of lymph nodes only ever approximate the cellular content of the node, a consistent aspiration technique was applied between patients and we could not attribute these differences to trivial anomalies in the cellular harvest of lymphomatous lymph nodes, which readily exfoliate cells. Rather, we attributed this observation to cellular effacement of the normal lymphoid tissue by neoplastic B or T cells.

The expression pattern of Helios was also unanticipated. Applied as a marker of thymic Tregs in our study [100], the high frequency of its expression in a number of TCL and two BCL cases suggested that neoplastic lymphocytes may also express this transcription factor, especially those of T cell origin. This intriguing observation accords with similar findings in human TCL, in which over-expression of non-DNA-binding, dominant negative isoforms of Helios is thought to be involved in disease progression [101]. Indeed, transgenic expression of short Helios isoforms in mice promotes the development of TCL [102], while ectopic expression of full-length Helios in B cells promotes BCL [103], suggesting a potential pathogenic role in both immunophenotypes. It is tempting to speculate that deregulated expression of Helios may also be implicated in a minority of BCLs and some TCLs in dogs. Insufficient data were available to allow survival analysis of the Helios^high^ group, but this could be a rewarding avenue for future research; furthermore, immunocytochemical staining could be employed to confirm expression of Helios by the malignant T cells. In the light of these observations, Helios was used as a surrogate marker for thymic Tregs only in the BCL dogs.

Review of the initial flow cytometric data prompted us to adopt a cascaded gating approach in subsequent analysis of the BCL cases, in which sequential CD5^+^ and CD4^+^ gates were applied to identify the resident T cells. By ‘normalizing’ the data in this manner, we endeavored to remove the dilutional influence of the neoplastic B cells. We found no significant difference in the frequency of FOXP3^+^ cells between the BCL and two control groups, when expressed either as a proportion of CD5^+^ or of CD5^+^CD4^+^ T cells. This observation contrasts with both a comparison of the cellular phenotype of lymph nodes in human patients with Hodgkin lymphoma and RH [104], and a recent study by Mitchell *et al*, who examined the cytotoxic T cell response in dogs with multicentric BCL treated with doxorubicin: the mean initial frequency of Tregs was approximately four-fold higher in Mitchell's study than in ours [105]. While this disparity is difficult to explain given the commonality of the species and disease, there were some notable differences between the studies. Thus, Mitchell *et al* harvested excisional biopsies, which represent a more accurate sampling technique than FNAs, but recruited only five dogs with BCL in total. We speculate that the five dogs included in their study represented a Treg^high^ phenotype, reminiscent of the highest-expressing patients in our own work, but that the pattern of expression is generally more heterogeneous among all dogs with BCL. Furthermore, for ethical and legal reasons we were unable to sample the lymph nodes of healthy animals; our comparisons were thus restricted to dogs with non-neoplastic lymphadenomegaly and infiltration of the lymph nodes by another round cell tumor, both of which could have harbored higher frequencies of Tregs than healthy dogs. Indeed, this notion of non-malignant expansion of Tregs reconciles with the known activation of these cells in the context of inflammatory microenvironments [106].

Among the populations of cytotoxic cells within a tumor, CD8^+^ T cells play a prominent role in the elimination of neoplastic cells [107]. Conventional wisdom places them on the other side of a balancing act with Tregs and additional regulatory cells in solid cancers [108]. We thus explored the possibility of a perturbation of the relative frequencies of CD8^+^ and FOXP3^+^ T cells in BCL, but found no evidence of a deficit of the former when compared to the control groups. Rather, the ratio of CD8^+^ to regulatory T cells tended to be higher in the lymph nodes of the BCL than RH dogs, especially when thymic Tregs were identified by means of Helios expression. While Helios has been criticized as a marker for thymic Tregs [109], it is still generally regarded to be a valid surrogate for this population of regulatory cells [110]. Future studies could also examine the cross-reactivity of available mAbs against neuropilin-1, considered by some to be a superior marker [111] and implicated in Treg-mediated suppression within certain cancer microenvironments [112]. Taken together, these data provided no support for a gross numerical deficit of CD8^+^ T cells in canine BCL, but further work will be required to explore possible functional deficits of these critical cells.

Despite the lack of a significant difference in overall T cell FOXP3 expression between the BCL and control groups, FOXP3 nevertheless emerged as an independent negative prognostic factor in this study. This apparent paradox was likely to reflect the very different microenvironments of B cell neoplasia and lymphoid hyperplasia and the role of Tregs within them: in the context of cancer, Tregs have traditionally been viewed as having a pathogenic influence [22], [23], [42], co-opted by the cancer to help thwart anti-neoplastic immune responses [113], while in the context of RH, Tregs are likely to serve a beneficial role by limiting over-exuberant inflammatory responses and collateral tissue damage, adapting their suppressive program to the prevailing milieu [106]. While our data reconciled with this view of Tregs as being ‘bad’ in the context of canine BCL, aligning with a plethora of studies of solid tissue cancers in humans and other species [22], [23], [42] and some studies of human NHL [27]–[31], it was at odds with a proportion of studies of human lymphoma that have suggested the exact opposite – that Tregs are actually part of the anti-neoplastic defenses [32]–[40], presumably because they are able to draw on their armamentarium of suppressive mechanisms to neutralize or kill neoplastic T and B cells, as well as a host of innate immune cells subverted by the lymphoma. The bewildering complexity of the microenvironment in both solid tissue and hemolymphatic tumors is only now beginning to be elucidated [16]–[20] and we speculate that Tregs may have divergent and dynamic roles in this context that are predicated on both micro-anatomical and temporal factors. Whether FOXP3^+^ Tregs play a negative prognostic role in all canine BCL subtypes remains to be determined, but they appeared to be deleterious in the context of the cohort of dogs we examined. High Helios and low MHC II expression were also of prognostic significance in this study. We suggest that the Helios result reflects its predominant co-localization with FOXP3 in thymic Tregs, while the MHC II result reconciles with studies of both human [114]–[116] and canine [117] BCL patients, in which low-level expression of this molecule is thought to compromise the activation of T cell responses against the neoplastic B cells and thus shorten survival. Interestingly, the current study revealed an impact of MHC II expression on TTR alone, but differences in patients and study design were likely to account for this discrepancy from existing publications.

In common with all clinical research of an exploratory nature, this study suffered a number of limitations. Staging was not complete in every case and our end-point variables (TTR, PFS and OS) were defined by owners' and attending veterinarians' observations of clinical signs and lymph node size. While diligent follow-up of cases was undertaken, the pragmatic nature of clinical veterinary practice meant that these values were likely to be best estimates only. Furthermore, the cohort of BCL patients examined was uncontrolled and relatively small, incorporating a number of different treatment regimens. We chose not to censor dogs that had received rescue therapy, instead incorporating them in the analysis of OS to maximize power. Nevertheless, despite the heterogeneity of the cohort, we were able to demonstrate that frequency of FOXP3 expression was an independent prognostic factor. Future studies of carefully stratified canine lymphoma patients treated with the same chemotherapy protocol are planned to confirm the results of the current work, which must be considered preliminary until they are prospectively validated in this manner.

In summary, we have presented evidence suggesting that FOXP3^+^ Tregs have a negative prognostic impact in canine multicentric BCL. We suggest that dogs with BCL, in common with human NHL patients, harbor a spectrum of interacting regulatory cells within the neoplastic lymph nodes, including thymic Tregs. We present further evidence of similarities between canine BCL and human NHL, thus helping to vindicate this large animal model of lymphoid cancer. There is an increasing need to find new ways of modulating the immune system as an integral part of multimodal veterinary cancer therapy. Regulatory T cells are an obvious candidate in such therapeutic endeavors, since this work suggests that they play a decisive role in the pathogenesis of canine BCL.

## Supporting Information

Figure S1 **Cytological characteristics of representative B cell lymphoma, T cell lymphoma, reactive hyperplasia and mast cell tumor cases.** Smears of fine needle aspirates were stained with modified Wright's stain and examined by a board-certified clinical pathologist in each case to reach a cytological diagnosis. A representative image of each group is presented (100x oil immersion lens; bar = 10 µm). (A) Diffuse large B cell lymphoma (DLBCL), characterized by a dominance of medium-to-large lymphocytes with immature chromatin and prominent nucleoli. (B) Peripheral T cell lymphoma (PTCL), characterized by a dominance of medium-to-large lymphocytes with eccentric and occasionally indented nuclei, smooth chromatin and multiple nucleoli. (C) Reactive hyperplasia (RH), characterized by a mixed population of lymphocytes with predominance of small lymphocytes, and increased numbers of medium-to-large lymphocytes, plasma cells and Mott cells. (D) Draining lymph node of a mast cell tumor (MCT), characterized by numerous moderately-to-well granulated mast cells, occasional eosinophils and a mixed lymphocyte population, consistent with a metastatic mast cell tumor.
(TIF)

Figure S2 **Younger dogs, those treated with CHOP and those ‘rescued’ show longer survival in B cell lymphoma.** (A) Dogs with B cell lymphoma that were less than or equal to the median age of the group (112 months) had longer progression-free survival (PFS: median 174 *vs* 74 days; median ratio = 0.43, p = 0.006) but not overall survival (OS, p = 0.11; data not shown); time to remission (TTR) was also no different between younger and older dogs (p = 0.57; data not shown). The significance of age on PFS remained in the multivariable regression model, demonstrating that it was an independent prognostic factor in this cohort of dogs. (B) CHOP chemotherapy was associated with longer PFS than the other treatments in the BCL group (CHOP, median 175 days; Other [including COP (n = 3), cytarabine, L-asparaginase, lomustine (n = 3) and prednisolone alone (n = 1)], median 45 days; p = 0.01). However, when interrogated in multivariable analysis, the variable ‘protocol’ no longer remained significant after accounting for age, reflecting the younger mean age of dogs treated with CHOP than ‘Other’ protocols (93 *versus* 123 months; p = 0.05). (C) Dogs receiving rescue therapy had longer OS (median 322 days) than those not receiving rescue therapy (174 days, median ratio 0.54; p = 0.049).
(TIF)

Table S1 **Cytomorphological criteria for the assessment of lymphoma cases.**
(DOC)

Table S2 **Signalment, therapy and immunophenotype of B cell lymphoma dogs.** Abbreviations: mo, months; m, male; f, female; n, neutered; e, entire; ND, not determined; chemotherapy agents: CHOP, protocol in which cyclophosphamide (C), doxorubicin (H), vincristine (O) and prednisolone (P) are administered; COP, protocol in which cyclophosphamide (C), vincristine (O) and prednisolone (P) are administered; L, lomustine; Cy, cytosine arabinoside; Ap, L-asparaginase; Chl, chlorambucil; VCAA, protocol in which vincristine (V), cyclophosphamide (C), L-asparaginase (A) and doxorubicin (A) are administered; LMP, protocol in which chlorambucil (L), methotrexate (M) and prednisolone (P) are administered; DMAC, protocol in which dexamethasone (D), melphelan (M), actinomycin-D (A) and cytosine arabinoside (C) are administered; Ma, masitinib; Vb, vinblastine; Pr, procarbazine; -, no rescue therapy administered (Rescue therapy) or remission not achieved (TTR); +, no progression (PFS) or alive at conclusion of study (OS) and therefore censored from survival analysis. Notes: The immunophenotype lists the per cent positive staining for the listed antigen; ^1^: these cases were classified as B cell lymphomas with aberrant CD5 expression.
(DOC)

Table S3 **Signalment, therapy and immunophenotype of T cell lymphoma dogs.** Abbreviations: mo, months; m, male; f, female; n, neutered; e, entire; ND, not determined; chemotherapy agents: see Table S2 and Dex, dexamethasone; -, no rescue therapy administered (Rescue therapy) or remission not achieved (TTR); +, no progression (PFS) or alive at conclusion of study (OS) and therefore censored from survival analysis. Notes: The immunophenotype lists the per cent positive staining for the listed antigen.
(DOC)

Table S4 **Disease subtype and signalment of reactive hyperplasia dogs.** Abbreviations: mo, months; m, male; f, female; n, neutered; e, entire.
(DOC)

Table S5 **Signalment of mast cell tumor dogs.** Abbreviations: mo, months; f, female; n, neutered; e, entire.
(DOC)

## References

[1] Guerard EJ, Bishop MR (2012) Overview of non-Hodgkin's lymphoma. Dis Mon 58: 208–218. 22449369 10.1016/j.disamonth.2012.01.010

[2] Shankland KR, Armitage JO, Hancock BW (2012) Non-Hodgkin lymphoma. Lancet 380: 848–857. 22835603 10.1016/S0140-6736(12)60605-9

[3] UK CR (2013) Cancer Statistics Report: Non-Hodgkin Lymphoma.

[4] O'Connor OA, Toner LE, Vrhovac R, Budak-Alpdogan T, Smith EA, et al (2005) Comparative animal models for the study of lymphohematopoietic tumors: strengths and limitations of present approaches. Leuk Lymphoma 46: 973–992. 16019548 10.1080/10428190500083193

[5] Head E (2013) A canine model of human aging and Alzheimer's disease. Biochim Biophys Acta 1832: 1384–1389. 23528711 10.1016/j.bbadis.2013.03.016 PMC3937962

[6] Potschka H, Fischer A, von Ruden EL, Hulsmeyer V, Baumgartner W (2013) Canine epilepsy as a translational model? Epilepsia 54: 571–579. 23506100 10.1111/epi.12138

[7] Ranieri G, Gadaleta CD, Patruno R, Zizzo N, Daidone MG, et al (2013) A model of study for human cancer: Spontaneous occurring tumors in dogs. Biological features and translation for new anticancer therapies. Crit Rev Oncol Hematol 88: 187–197. 23561333 10.1016/j.critrevonc.2013.03.005

[8] Tsai KL, Clark LA, Murphy KE (2007) Understanding hereditary diseases using the dog and human as companion model systems. Mamm Genome 18: 444–451. 17653794 10.1007/s00335-007-9037-1 PMC1998873

[9] Gordon I, Paoloni M, Mazcko C, Khanna C (2009) The Comparative Oncology Trials Consortium: using spontaneously occurring cancers in dogs to inform the cancer drug development pathway. PLoS Med 6: e1000161. 19823573 10.1371/journal.pmed.1000161 PMC2753665

[10] Paoloni M, Khanna C (2008) Translation of new cancer treatments from pet dogs to humans. Nat Rev Cancer 8: 147–156. 18202698 10.1038/nrc2273

[11] Marconato L, Gelain ME, Comazzi S (2013) The dog as a possible animal model for human non-Hodgkin lymphoma: a review. Hematol Oncol 31: 1–9. 22674797 10.1002/hon.2017

[12] Frantz AM, Sarver AL, Ito D, Phang TL, Karimpour-Fard A, et al (2013) Molecular profiling reveals prognostically significant subtypes of canine lymphoma. Vet Pathol 50: 693–703. 23125145 10.1177/0300985812465325 PMC4683027

[13] Mooney M, Bond J, Monks N, Eugster E, Cherba D, et al (2013) Comparative RNA-Seq and microarray analysis of gene expression changes in B-cell lymphomas of Canis familiaris. PLoS One 8: e61088. 23593398 10.1371/journal.pone.0061088 PMC3617154

[14] Richards KL, Motsinger-Reif AA, Chen HW, Fedoriw Y, Fan C, et al (2013) Gene Profiling of Canine B-Cell Lymphoma Reveals Germinal Center and Postgerminal Center Subtypes with Different Survival Times, Modeling Human DLBCL. Cancer Res 73: 5029–5039. 23783577 10.1158/0008-5472.CAN-12-3546 PMC3755352

[15] Su Y, Nielsen D, Zhu L, Richards K, Suter S, et al (2013) Gene selection and cancer type classification of diffuse large-B-cell lymphoma using a bivariate mixture model for two-species data. Hum Genomics 7: 2. 23289441 10.1186/1479-7364-7-2 PMC3618031

[16] Schiavoni G, Gabriele L, Mattei F (2013) The tumor microenvironment: a pitch for multiple players. Front Oncol 3: 90. 23616948 10.3389/fonc.2013.00090 PMC3628362

[17] Becker JC, Andersen MH, Schrama D, Thor Straten P (2013) Immune-suppressive properties of the tumor microenvironment. Cancer Immunol Immunother 62: 1137–1148. 23666510 10.1007/s00262-013-1434-6 PMC11029603

[18] Gribben JG (2010) Implications of the tumor microenvironment on survival and disease response in follicular lymphoma. Curr Opin Oncol 22: 424–430. 20679770 10.1097/CCO.0b013e32833d5938

[19] Coupland SE (2011) The challenge of the microenvironment in B-cell lymphomas. Histopathology 58: 69–80. 21261684 10.1111/j.1365-2559.2010.03706.x

[20] Fridman WH, Dieu-Nosjean MC, Pages F, Cremer I, Damotte D, et al (2013) The immune microenvironment of human tumors: general significance and clinical impact. Cancer Microenviron 6: 117–122. 23108700 10.1007/s12307-012-0124-9 PMC3717061

[21] Garden OA, Pinheiro D, Cunningham F (2011) All creatures great and small: regulatory T cells in mice, humans, dogs and other domestic animal species. Int Immunopharmacol 11: 576–588. 21093606 10.1016/j.intimp.2010.11.003

[22] Gallimore A, Godkin A (2008) Regulatory T cells and tumour immunity - observations in mice and men. Immunology 123: 157–163. 18067556 10.1111/j.1365-2567.2007.02748.x PMC2433304

[23] Oleinika K, Nibbs RJ, Graham GJ, Fraser AR (2013) Suppression, subversion and escape: the role of regulatory T cells in cancer progression. Clin Exp Immunol 171: 36–45. 23199321 10.1111/j.1365-2249.2012.04657.x PMC3530093

[24] Dobrzanski MJ (2013) Expanding roles for CD4 T cells and their subpopulations in tumor immunity and therapy. Front Oncol 3: 63. 23533029 10.3389/fonc.2013.00063 PMC3607796

[25] deLeeuw RJ, Kost SE, Kakal JA, Nelson BH (2012) The prognostic value of FoxP3+ tumor-infiltrating lymphocytes in cancer: a critical review of the literature. Clin Cancer Res 18: 3022–3029. 22510350 10.1158/1078-0432.CCR-11-3216

[26] Lindqvist CA, Loskog AS (2012) T regulatory cells in B-cell malignancy - tumour support or kiss of death? Immunology 135: 255–260. 22112044 10.1111/j.1365-2567.2011.03539.x PMC3372741

[27] Marshall NA, Christie LE, Munro LR, Culligan DJ, Johnston PW, et al (2004) Immunosuppressive regulatory T cells are abundant in the reactive lymphocytes of Hodgkin lymphoma. Blood 103: 1755–1762. 14604957 10.1182/blood-2003-07-2594

[28] Yang ZZ, Novak AJ, Ziesmer SC, Witzig TE, Ansell SM (2006) Attenuation of CD8^+^ T-cell function by CD4^+^CD25^+^ regulatory T cells in B-cell non-Hodgkin's lymphoma. Cancer Res 66: 10145–10152. 17047079 10.1158/0008-5472.CAN-06-1822 PMC2680600

[29] Yang ZZ, Novak AJ, Stenson MJ, Witzig TE, Ansell SM (2006) Intratumoral CD4^+^CD25^+^ regulatory T-cell-mediated suppression of infiltrating CD4^+^ T cells in B-cell non-Hodgkin lymphoma. Blood 107: 3639–3646. 16403912 10.1182/blood-2005-08-3376 PMC1895773

[30] Yang ZZ, Novak AJ, Ziesmer SC, Witzig TE, Ansell SM (2009) Malignant B cells skew the balance of regulatory T cells and TH17 cells in B-cell non-Hodgkin's lymphoma. Cancer Res 69: 5522–5530. 19509224 10.1158/0008-5472.CAN-09-0266 PMC2764404

[31] Schreck S, Friebel D, Buettner M, Distel L, Grabenbauer G, et al (2009) Prognostic impact of tumour-infiltrating Th2 and regulatory T cells in classical Hodgkin lymphoma. Hematol Oncol 27: 31–39. 18924115 10.1002/hon.878

[32] Alvaro T, Lejeune M, Salvado MT, Bosch R, Garcia JF, et al (2005) Outcome in Hodgkin's lymphoma can be predicted from the presence of accompanying cytotoxic and regulatory T cells. Clin Cancer Res 11: 1467–1473. 15746048 10.1158/1078-0432.CCR-04-1869

[33] Carreras J, Lopez-Guillermo A, Fox BC, Colomo L, Martinez A, et al (2006) High numbers of tumor-infiltrating FOXP3-positive regulatory T cells are associated with improved overall survival in follicular lymphoma. Blood 108: 2957–2964. 16825494 10.1182/blood-2006-04-018218

[34] Kelley TW, Pohlman B, Elson P, Hsi ED (2007) The ratio of FOXP3^+^ regulatory T cells to granzyme B^+^ cytotoxic T/NK cells predicts prognosis in classical Hodgkin lymphoma and is independent of bcl-2 and MAL expression. Am J Clin Pathol 128: 958–965. 18024321 10.1309/NB3947K383DJ0LQ2

[35] Lee NR, Song EK, Jang KY, Choi HN, Moon WS, et al (2008) Prognostic impact of tumor infiltrating FOXP3 positive regulatory T cells in diffuse large B-cell lymphoma at diagnosis. Leuk Lymphoma 49: 247–256. 18231910 10.1080/10428190701824536

[36] Tzankov A, Meier C, Hirschmann P, Went P, Pileri SA, et al (2008) Correlation of high numbers of intratumoral FOXP3^+^ regulatory T cells with improved survival in germinal center-like diffuse large B-cell lymphoma, follicular lymphoma and classical Hodgkin's lymphoma. Haematologica 93: 193–200. 18223287 10.3324/haematol.11702

[37] Wahlin BE, Aggarwal M, Montes-Moreno S, Gonzalez LF, Roncador G, et al (2010) A unifying microenvironment model in follicular lymphoma: outcome is predicted by programmed death-1–positive, regulatory, cytotoxic, and helper T cells and macrophages. Clin Cancer Res 16: 637–650. 20068089 10.1158/1078-0432.CCR-09-2487

[38] Koreishi AF, Saenz AJ, Persky DO, Cui H, Moskowitz A, et al (2010) The role of cytotoxic and regulatory T cells in relapsed/refractory Hodgkin lymphoma. Appl Immunohistochem Mol Morphol 18: 206–211. 20065852 10.1097/PAI.0b013e3181c7138b PMC3260943

[39] Felcht M, Heck M, Weiss C, Becker JC, Dippel E, et al (2012) Expression of the T-cell regulatory marker FOXP3 in primary cutaneous large B-cell lymphoma tumour cells. Br J Dermatol 167: 348–358. 22512270 10.1111/j.1365-2133.2012.10987.x

[40] Dehghani M, Sharifpour S, Amirghofran Z, Zare HR (2012) Prognostic significance of T cell subsets in peripheral blood of B cell non-Hodgkin's lymphoma patients. Med Oncol 29: 2364–2371. 22307765 10.1007/s12032-012-0176-1

[41] Koch K, Hoster E, Unterhalt M, Ott G, Rosenwald A, et al (2012) The composition of the microenvironment in follicular lymphoma is associated with the stage of the disease. Hum Pathol 43: 2274–2281. 22795355 10.1016/j.humpath.2012.03.025

[42] Lindau D, Gielen P, Kroesen M, Wesseling P, Adema GJ (2013) The immunosuppressive tumour network: myeloid-derived suppressor cells, regulatory T cells and natural killer T cells. Immunology 138: 105–115. 23216602 10.1111/imm.12036 PMC3575763

[43] Whiteside TL, Schuler P, Schilling B (2012) Induced and natural regulatory T cells in human cancer. Expert Opin Biol Ther 12: 1383–1397. 22849383 10.1517/14712598.2012.707184 PMC3730844

[44] Tanchot C, Terme M, Pere H, Tran T, Benhamouda N, et al (2013) Tumor-infiltrating regulatory T cells: phenotype, role, mechanism of expansion in situ and clinical significance. Cancer Microenviron 6: 147–157. 23104434 10.1007/s12307-012-0122-y PMC3717062

[45] Adeegbe DO, Nishikawa H (2013) Natural and induced T regulatory cells in cancer. Front Immunol 4: 190. 23874336 10.3389/fimmu.2013.00190 PMC3708155

[46] Pinheiro D, Singh Y, Grant CR, Appleton RC, Sacchini F, et al (2011) Phenotypic and functional characterization of a CD4^+^CD25^high^FOXP3^high^ regulatory T-cell population in the dog. Immunology 132: 111–122. 20880379 10.1111/j.1365-2567.2010.03346.x PMC3015081

[47] Wilkerson MJ, Dolce K, Koopman T, Shuman W, Chun R, et al (2005) Lineage differentiation of canine lymphoma/leukemias and aberrant expression of CD molecules. Vet Immunol Immunopathol 106: 179–196. 15963817 10.1016/j.vetimm.2005.02.020

[48] Vail DM, Michels GM, Khanna C, Selting KA, London CA (2010) Response evaluation criteria for peripheral nodal lymphoma in dogs (v1.0)–a Veterinary Cooperative Oncology Group (VCOG) consensus document. Vet Comp Oncol 8: 28–37. 20230579 10.1111/j.1476-5829.2009.00200.x

[49] Modiano JF, Breen M, Burnett RC, Parker HG, Inusah S, et al (2005) Distinct B-cell and T-cell lymphoproliferative disease prevalence among dog breeds indicates heritable risk. Cancer Res 65: 5654–5661. 15994938 10.1158/0008-5472.CAN-04-4613

[50] Lurie DM, Milner RJ, Suter SE, Vernau W (2008) Immunophenotypic and cytomorphologic subclassification of T-cell lymphoma in the boxer breed. Vet Immunol Immunopathol 125: 102–110. 18579219 10.1016/j.vetimm.2008.05.009

[51] Ponce F, Marchal T, Magnol JP, Turinelli V, Ledieu D, et al (2010) A morphological study of 608 cases of canine malignant lymphoma in France with a focus on comparative similarities between canine and human lymphoma morphology. Vet Pathol 47: 414–433. 20472804 10.1177/0300985810363902

[52] Pastor M, Chalvet-Monfray K, Marchal T, Keck G, Magnol JP, et al (2009) Genetic and environmental risk indicators in canine non-Hodgkin's lymphomas: breed associations and geographic distribution of 608 cases diagnosed throughout France over 1 year. J Vet Intern Med 23: 301–310. 19192140 10.1111/j.1939-1676.2008.0255.x

[53] Greenlee PG, Filippa DA, Quimby FW, Patnaik AK, Calvano SE, et al (1990) Lymphomas in dogs. A morphologic, immunologic, and clinical study. Cancer 66: 480–490. 2364361 10.1002/1097-0142(19900801)66:3<480::aid-cncr2820660314>3.0.co;2-x

[54] Fournel-Fleury C, Ponce F, Felman P, Blavier A, Bonnefont C, et al (2002) Canine T-cell lymphomas: a morphological, immunological, and clinical study of 46 new cases. Vet Pathol 39: 92–109. 12102223 10.1354/vp.39-1-92

[55] Fournel-Fleury C, Magnol JP, Bricaire P, Marchal T, Chabanne L, et al (1997) Cytohistological and immunological classification of canine malignant lymphomas: comparison with human non-Hodgkin's lymphomas. J Comp Pathol 117: 35–59. 9263843 10.1016/s0021-9975(97)80065-5

[56] Ponce F, Magnol JP, Ledieu D, Marchal T, Turinelli V, et al (2004) Prognostic significance of morphological subtypes in canine malignant lymphomas during chemotherapy. Vet J 167: 158–166. 14975390 10.1016/j.tvjl.2003.10.009

[57] Mey U, Hitz F, Lohri A, Pederiva S, Taverna C, et al (2012) Diagnosis and treatment of diffuse large B-cell lymphoma. Swiss Med Wkly 142: w13511. 22290632 10.4414/smw.2012.13511

[58] Das DK (1999) Value and limitations of fine-needle aspiration cytology in diagnosis and classification of lymphomas: A review. Diagn Cytopathol 21: 240–249. 10495316 10.1002/(sici)1097-0339(199910)21:4<240::aid-dc3>3.0.co;2-z

[59] Landgren O, Porwit MacDonald A, Tani E, Czader M, Grimfors G, et al (2004) A prospective comparison of fine-needle aspiration cytology and histopathology in the diagnosis and classification of lymphomas. Hematol J 5: 69–76. 14745433 10.1038/sj.thj.6200316

[60] Vigliar E, Cipullo C, Todaro P, Giuffre G, Pepe S (2012) Fine needle cytology, infectious diseases and non-Hodgkin lymphoma. Infez Med 20 Suppl 3: 39–42. 23069693

[61] Aresu L, Martini V, Rossi F, Vignoli M, Sampaolo M, et al.. (2013) Canine indolent and aggressive lymphoma: clinical spectrum with histologic correlation. Vet Comp Oncol. doi: 10.1111/vco.12048. 10.1111/vco.12048 23782432

[62] Valli VE, Kass PH, Myint MS, Scott F (2013) Canine lymphomas: association of classification type, disease stage, tumor subtype, mitotic rate, and treatment with survival. Vet Pathol 50: 738–748. 23444036 10.1177/0300985813478210

[63] Valli VE, San Myint M, Barthel A, Bienzle D, Caswell J, et al (2011) Classification of canine malignant lymphomas according to the World Health Organization criteria. Vet Pathol 48: 198–211. 20861499 10.1177/0300985810379428

[64] Marconato L (2011) The staging and treatment of multicentric high-grade lymphoma in dogs: a review of recent developments and future prospects. Vet J 188: 34–38. 20627636 10.1016/j.tvjl.2010.04.027

[65] Chun R (2009) Lymphoma: which chemotherapy protocol and why? Top Companion Anim Med 24: 157–162. 19732735 10.1053/j.tcam.2009.03.003

[66] Liu P, Jaffar J, Hellstrom I, Hellstrom KE (2010) Administration of cyclophosphamide changes the immune profile of tumor-bearing mice. J Immunother 33: 53–59. 19952956 10.1097/CJI.0b013e3181b56af4 PMC2811714

[67] Rico MJ, Rozados VR, Mainetti LE, Zacarias Fluck MF, Matar P, et al (2012) Regulatory T cells but not NKT I cells are modulated by a single low-dose cyclophosphamide in a B cell lymphoma tumor-model. Exp Oncol 34: 38–42. 22453147

[68] Mao R, Xiao W, Liu H, Chen B, Yi B, et al (2013) Systematic evaluation of 640 FDA drugs for their effect on CD4^+^Foxp3^+^ regulatory T cells using a novel cell-based high throughput screening assay. Biochem Pharmacol 85: 1513–1524. 23537702 10.1016/j.bcp.2013.03.013

[69] Zhao J, Cao Y, Lei Z, Yang Z, Zhang B, et al (2010) Selective depletion of CD4^+^CD25^+^Foxp3^+^ regulatory T cells by low-dose cyclophosphamide is explained by reduced intracellular ATP levels. Cancer Res 70: 4850–4858. 20501849 10.1158/0008-5472.CAN-10-0283

[70] Camisaschi C, Filipazzi P, Tazzari M, Casati C, Beretta V, et al (2013) Effects of cyclophosphamide and IL-2 on regulatory CD4^+^ T cell frequency and function in melanoma patients vaccinated with HLA-class I peptides: impact on the antigen-specific T cell response. Cancer Immunol Immunother 62: 897–908. 23589107 10.1007/s00262-013-1397-7 PMC3634989

[71] Zen M, Canova M, Campana C, Bettio S, Nalotto L, et al (2011) The kaleidoscope of glucorticoid effects on immune system. Autoimmun Rev 10: 305–310. 21224015 10.1016/j.autrev.2010.11.009

[72] Burton JH, Mitchell L, Thamm DH, Dow SW, Biller BJ (2011) Low-dose cyclophosphamide selectively decreases regulatory T cells and inhibits angiogenesis in dogs with soft tissue sarcoma. J Vet Intern Med 25: 920–926. 21736624 10.1111/j.1939-1676.2011.0753.x

[73] Moniuszko M, Bodzenta-Lukaszyk A, Dabrowska M (2010) Effects of oral glucocorticoid therapy on CD4^+^CD25^+^CD127^−^ and CD4^+^CD25^high^ T cell levels in asthmatic patients. Inflammation 33: 415–420. 20300815 10.1007/s10753-010-9200-9

[74] Sbiera S, Dexneit T, Reichardt SD, Michel KD, van den Brandt J, et al (2011) Influence of short-term glucocorticoid therapy on regulatory T cells in vivo. PLoS One 6: e24345. 21912688 10.1371/journal.pone.0024345 PMC3166315

[75] Armand P, Welch S, Kim HT, LaCasce AS, Jacobsen ED, et al (2013) Prognostic factors for patients with diffuse large B cell lymphoma and transformed indolent lymphoma undergoing autologous stem cell transplantation in the positron emission tomography era. Br J Haematol 160: 608–617. 23278720 10.1111/bjh.12176

[76] A predictive model for aggressive non-Hodgkin's lymphoma. The International Non-Hodgkin's Lymphoma Prognostic Factors Project. N Engl J Med 329: 987–994. 8141877 10.1056/NEJM199309303291402

[77] Fields PA, Linch DC (2012) Treatment of the elderly patient with diffuse large B cell lymphoma. Br J Haematol 157: 159–170. 22463486 10.1111/j.1365-2141.2011.09011.x

[78] MacEwen EG, Hayes AA, Mooney S, Patnaik A, Kurzman I, et al (1985) Levamisole as adjuvant to chemotherapy for canine lymphosarcoma. J Biol Response Mod 4: 427–433. 3839843

[79] Keller ET, MacEwen EG, Rosenthal RC, Helfand SC, Fox LE (1993) Evaluation of prognostic factors and sequential combination chemotherapy with doxorubicin for canine lymphoma. J Vet Intern Med 7: 289–295. 8263847 10.1111/j.1939-1676.1993.tb01021.x

[80] Dobson JM, Blackwood LB, McInnes EF, Bostock DE, Nicholls P, et al (2001) Prognostic variables in canine multicentric lymphosarcoma. J Small Anim Pract 42: 377–384. 11518416 10.1111/j.1748-5827.2001.tb02485.x

[81] Teske E, van Heerde P, Rutteman GR, Kurzman ID, Moore PF, et al (1994) Prognostic factors for treatment of malignant lymphoma in dogs. J Am Vet Med Assoc 205: 1722–1728. 7744644

[82] Ruslander DA, Gebhard DH, Tompkins MB, Grindem CB, Page RL (1997) Immunophenotypic characterization of canine lymphoproliferative disorders. In Vivo 11: 169–172. 9179611

[83] Jagielski D, Lechowski R, Hoffmann-Jagielska M, Winiarczyk S (2002) A retrospective study of the incidence and prognostic factors of multicentric lymphoma in dogs (1998–2000). J Vet Med A Physiol Pathol Clin Med 49: 419–424. 12450190 10.1046/j.1439-0442.2002.00458.x

[84] Marconato L, Martini V, Aresu L, Sampaolo M, Valentini F, et al (2013) Assessment of bone marrow infiltration diagnosed by flow cytometry in canine large B cell lymphoma: Prognostic significance and proposal of a cut-off value. Vet J 197: 776–781. 23735731 10.1016/j.tvjl.2013.05.003

[85] Wiedemann AL, Charney SC, Barger AM, Schaeffer DJ, Kitchell BE (2005) Assessment of corticosteroid-induced alkaline phosphatase as a prognostic indicator in canine lymphoma. J Small Anim Pract 46: 185–190. 15835237 10.1111/j.1748-5827.2005.tb00309.x

[86] Weller RE, Theilen GH, Madewell BR (1982) Chemotherapeutic responses in dogs with lymphosarcoma and hypercalcemia. J Am Vet Med Assoc 181: 891–893. 6897239

[87] Miller AG, Morley PS, Rao S, Avery AC, Lana SE, et al (2009) Anemia is associated with decreased survival time in dogs with lymphoma. J Vet Intern Med 23: 116–122. 19138381 10.1111/j.1939-1676.2008.00210.x

[88] Perry JA, Thamm DH, Eickhoff J, Avery AC, Dow SW (2011) Increased monocyte chemotactic protein-1 concentration and monocyte count independently associate with a poor prognosis in dogs with lymphoma. Vet Comp Oncol 9: 55–64. 21303454 10.1111/j.1476-5829.2010.00235.x

[89] Price GS, Page RL, Fischer BM, Levine JF, Gerig TM (1991) Efficacy and toxicity of doxorubicin/cyclophosphamide maintenance therapy in dogs with multicentric lymphosarcoma. J Vet Intern Med 5: 259–262. 1748977 10.1111/j.1939-1676.1991.tb03131.x

[90] Khanna C, Lund EM, Redic KA, Hayden DW, Bell FW, et al (1998) Randomized controlled trial of doxorubicin versus dactinomycin in a multiagent protocol for treatment of dogs with malignant lymphoma. J Am Vet Med Assoc 213: 985–990. 9776993

[91] Carter RF, Harris CK, Withrow SJ, Valli VEO, Susaneck SJ (1987) Chemotherapy of canine lymphoma with histopathological correlation - doxorubicin alone compared to COP as first treatment regimen. J Am Anim Hosp Assoc 23: 587–598.

[92] Kol A, Marks SL, Skorupski KA, Kass PH, Guerrero T, et al.. (2013) Serial haemostatic monitoring of dogs with multicentric lymphoma. Vet Comp Oncol. doi: 10.1111/vco.12041. 10.1111/vco.12041 23710569

[93] Sato M, Yamzaki J, Goto-Koshino Y, Takahashi M, Fujino Y, et al (2013) The prognostic significance of minimal residual disease in the early phases of chemotherapy in dogs with high-grade B-cell lymphoma. Vet J 195: 319–324. 22909955 10.1016/j.tvjl.2012.07.013

[94] Webster JD, Dennis MM, Dervisis N, Heller J, Bacon NJ, et al (2011) Recommended guidelines for the conduct and evaluation of prognostic studies in veterinary oncology. Vet Pathol 48: 7–18. 20664014 10.1177/0300985810377187

[95] Mizuno T, Suzuki R, Umeki S, Okuda M (2009) Crossreactivity of antibodies to canine CD25 and Foxp3 and identification of canine CD4^+^CD25^+^Foxp3^+^ cells in canine peripheral blood. J Vet Med Sci 71: 1561–1568. 20046022 10.1292/jvms.001561

[96] Flammiger A, Weisbach L, Huland H, Tennstedt P, Simon R, et al (2013) High tissue density of FOXP3^+^ T cells is associated with clinical outcome in prostate cancer. Eur J Cancer 49: 1273–1279. 23266046 10.1016/j.ejca.2012.11.035

[97] Lee S, Cho EY, Park YH, Ahn JS, Im YH (2013) Prognostic impact of FOXP3 expression in triple-negative breast cancer. Acta Oncol 52: 73–81. 23075422 10.3109/0284186X.2012.731520

[98] Kryczek I, Liu R, Wang G, Wu K, Shu X, et al (2009) FOXP3 defines regulatory T cells in human tumor and autoimmune disease. Cancer Res 69: 3995–4000. 19383912 10.1158/0008-5472.CAN-08-3804

[99] Wang J, Ioan-Facsinay A, van der Voort EI, Huizinga TW, Toes RE (2007) Transient expression of FOXP3 in human activated nonregulatory CD4^+^ T cells. Eur J Immunol 37: 129–138. 17154262 10.1002/eji.200636435

[100] Thornton AM, Korty PE, Tran DQ, Wohlfert EA, Murray PE, et al (2010) Expression of Helios, an Ikaros transcription factor family member, differentiates thymic-derived from peripherally induced Foxp3^+^ T regulatory cells. J Immunol 184: 3433–3441. 20181882 10.4049/jimmunol.0904028 PMC3725574

[101] Fujii K, Ishimaru F, Nakase K, Tabayashi T, Kozuka T, et al (2003) Over-expression of short isoforms of Helios in patients with adult T-cell leukaemia/lymphoma. Br J Haematol 120: 986–989. 12648068 10.1046/j.1365-2141.2003.04216.x

[102] Zhang Z, Swindle CS, Bates JT, Ko R, Cotta CV, et al (2007) Expression of a non-DNA-binding isoform of Helios induces T-cell lymphoma in mice. Blood 109: 2190–2197. 17110463 10.1182/blood-2005-01-031930 PMC1801072

[103] Dovat S, Montecino-Rodriguez E, Schuman V, Teitell MA, Dorshkind K, et al (2005) Transgenic expression of Helios in B lineage cells alters B cell properties and promotes lymphomagenesis. J Immunol 175: 3508–3515. 16148093 10.4049/jimmunol.175.6.3508

[104] Hudnall SD, Betancourt E, Barnhart E, Patel J (2008) Comparative flow immunophenotypic features of the inflammatory infiltrates of Hodgkin lymphoma and lymphoid hyperplasia. Cytometry B Clin Cytom 74: 1–8. 18061945 10.1002/cyto.b.20376

[105] Mitchell L, Dow SW, Slansky JE, Biller BJ (2012) Induction of remission results in spontaneous enhancement of anti-tumor cytotoxic T-lymphocyte activity in dogs with B cell lymphoma. Vet Immunol Immunopathol 145: 597–603. 22293625 10.1016/j.vetimm.2012.01.006

[106] Chaudhry A, Rudensky AY (2013) Control of inflammation by integration of environmental cues by regulatory T cells. J Clin Invest 123: 939–944. 23454755 10.1172/JCI57175 PMC3582113

[107] Lanca T, Silva-Santos B (2012) The split nature of tumor-infiltrating leukocytes: Implications for cancer surveillance and immunotherapy. Oncoimmunology 1: 717–725. 22934263 10.4161/onci.20068 PMC3429575

[108] Lakshmi Narendra B, Eshvendar Reddy K, Shantikumar S, Ramakrishna S (2013) Immune system: a double-edged sword in cancer. Inflamm Res 62: 823–834. 23868500 10.1007/s00011-013-0645-9

[109] Akimova T, Beier UH, Wang L, Levine MH, Hancock WW (2011) Helios expression is a marker of T cell activation and proliferation. PLoS One 6: e24226. 21918685 10.1371/journal.pone.0024226 PMC3168881

[110] Onoe T, Kalscheuer H, Danzl N, Chittenden M, Zhao G, et al (2011) Human natural regulatory T cell development, suppressive function, and postthymic maturation in a humanized mouse model. J Immunol 187: 3895–3903. 21876039 10.4049/jimmunol.1100394 PMC3201793

[111] Lin X, Chen M, Liu Y, Guo Z, He X, et al (2013) Advances in distinguishing natural from induced Foxp3^+^ regulatory T cells. Int J Clin Exp Pathol 6: 116–123. 23329997 PMC3544233

[112] Hansen W, Hutzler M, Abel S, Alter C, Stockmann C, et al (2012) Neuropilin 1 deficiency on CD4^+^Foxp3^+^ regulatory T cells impairs mouse melanoma growth. J Exp Med 209: 2001–2016. 23045606 10.1084/jem.20111497 PMC3478934

[113] Malchow S, Leventhal DS, Nishi S, Fischer BI, Shen L, et al (2013) Aire-dependent thymic development of tumor-associated regulatory T cells. Science 339: 1219–1224. 23471412 10.1126/science.1233913 PMC3622085

[114] Rimsza LM, Farinha P, Fuchs DA, Masoudi H, Connors JM, et al (2007) HLA-DR protein status predicts survival in patients with diffuse large B-cell lymphoma treated on the MACOP-B chemotherapy regimen. Leuk Lymphoma 48: 542–546. 17454596 10.1080/10428190601078605

[115] Rimsza LM, Roberts RA, Miller TP, Unger JM, LeBlanc M, et al (2004) Loss of MHC class II gene and protein expression in diffuse large B-cell lymphoma is related to decreased tumor immunosurveillance and poor patient survival regardless of other prognostic factors: a follow-up study from the Leukemia and Lymphoma Molecular Profiling Project. Blood 103: 4251–4258. 14976040 10.1182/blood-2003-07-2365

[116] Veelken H, Vik Dannheim S, Schulte Moenting J, Martens UM, Finke J, et al (2007) Immunophenotype as prognostic factor for diffuse large B-cell lymphoma in patients undergoing clinical risk-adapted therapy. Ann Oncol 18: 931–939. 17395602 10.1093/annonc/mdm012

[117] Rao S, Lana S, Eickhoff J, Marcus E, Avery PR, et al (2011) Class II major histocompatibility complex expression and cell size independently predict survival in canine B-cell lymphoma. J Vet Intern Med 25: 1097–1105. 21781170 10.1111/j.1939-1676.2011.0767.x

