# Supplementary figures and images for: Dissecting the Regulatory Microenvironment of a Large Animal Model of Non-Hodgkin Lymphoma: Evidence of a Negative Prognostic Impact of FOXP3+ T Cells in Canine B Cell Lymphoma
Source: PLoS One. 2014 Aug 13;9(8):e105027. doi: 10.1371/journal.pone.0105027 (PMC4132014; doi:10.1371/journal.pone.0105027)

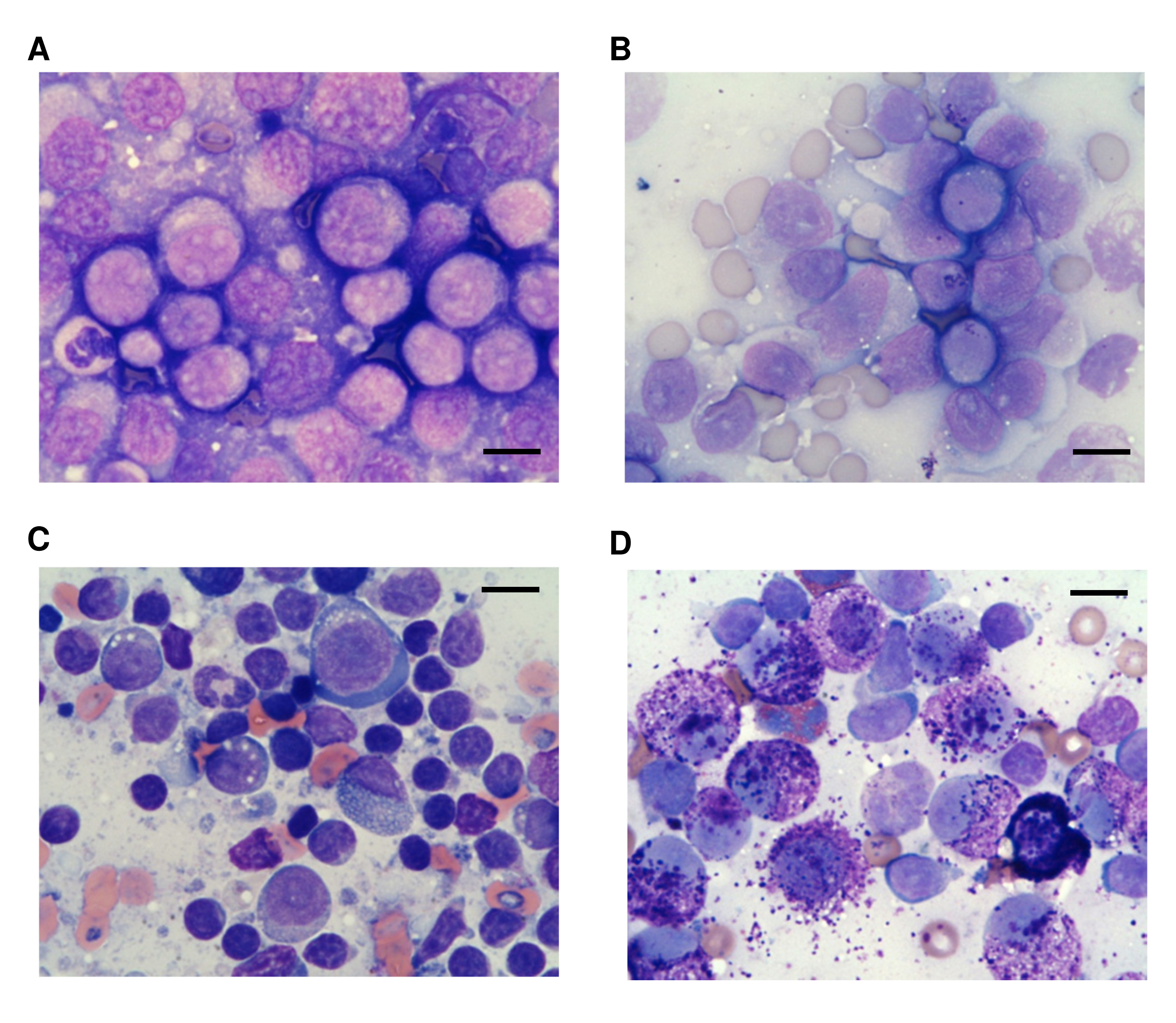

Supplement: Figure S1 — Cytological characteristics of representative B cell lymphoma, T cell lymphoma, reactive hyperplasia and mast cell tumor cases. Smears of fine needle aspirates were stained with modified Wright's stain and examined by a board-certified clinical pathologist in each case to reach a cytological diagnosis. A representative image of each group is presented (100x oil immersion lens; bar = 10 µm). (A) Diffuse large B cell lymphoma (DLBCL), characterized by a dominance of medium-to-large lymphocytes with immature chromatin and prominent nucleoli. (B) Peripheral T cell lymphoma (PTCL), characterized by a dominance of medium-to-large lymphocytes with eccentric and occasionally indented nuclei, smooth chromatin and multiple nucleoli. (C) Reactive hyperplasia (RH), characterized by a mixed population of lymphocytes with predominance of small lymphocytes, and increased numbers of medium-to-large lymphocytes, plasma cells and Mott cells. (D) Draining lymph node of a mast cell tumor (MCT), characterized by numerous moderately-to-well granulated mast cells, occasional eosinophils and a mixed lymphocyte population, consistent with a metastatic mast cell tumor. (TIF) [file pone.0105027.s001.tif]

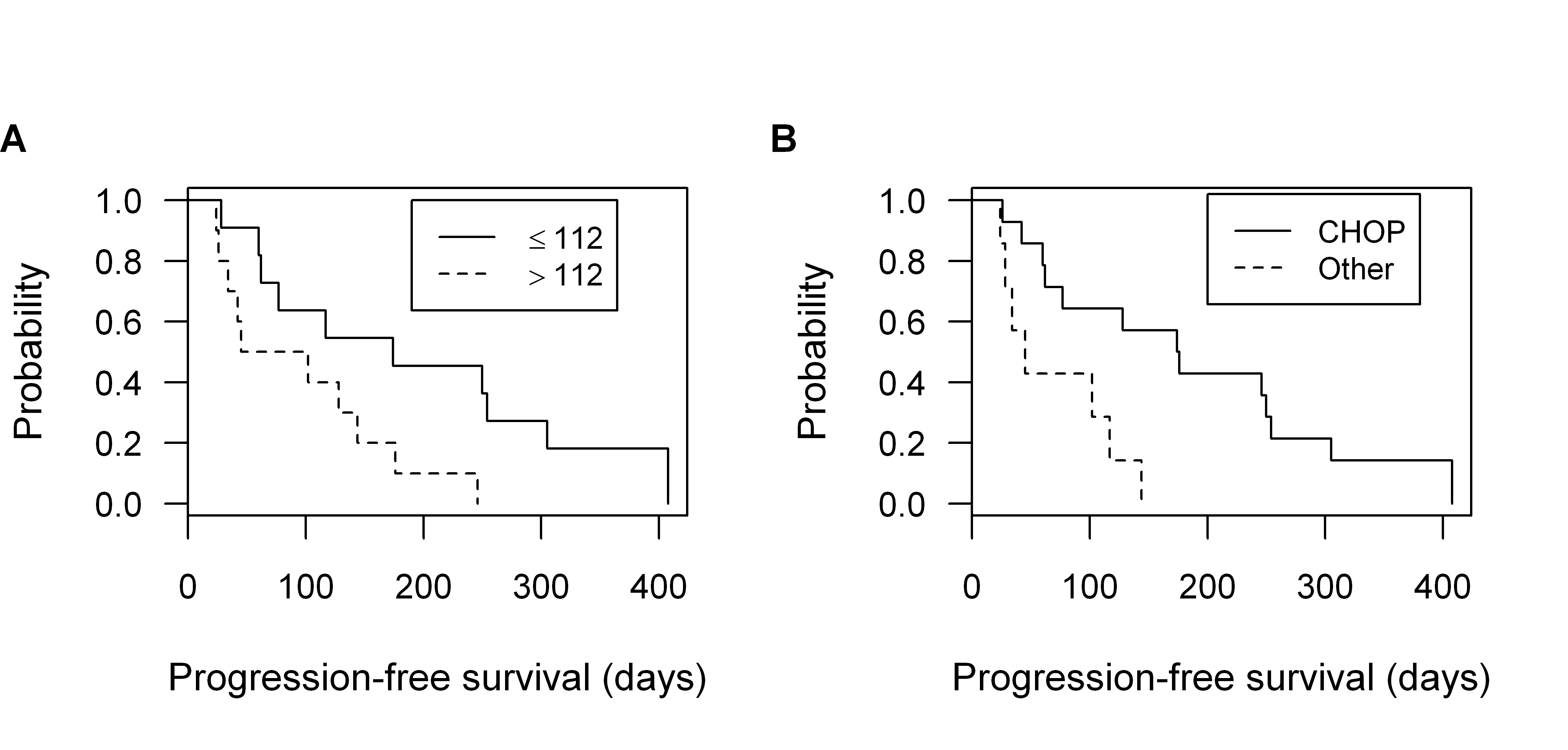

Supplement: Figure S2 — Younger dogs, those treated with CHOP and those ‘rescued’ show longer survival in B cell lymphoma. (A) Dogs with B cell lymphoma that were less than or equal to the median age of the group (112 months) had longer progression-free survival (PFS: median 174 vs 74 days; median ratio = 0.43, p = 0.006) but not overall survival (OS, p = 0.11; data not shown); time to remission (TTR) was also no different between younger and older dogs (p = 0.57; data not shown). The significance of age on PFS remained in the multivariable regression model, demonstrating that it was an independent prognostic factor in this cohort of dogs. (B) CHOP chemotherapy was associated with longer PFS than the other treatments in the BCL group (CHOP, median 175 days; Other [including COP (n = 3), cytarabine, L-asparaginase, lomustine (n = 3) and prednisolone alone (n = 1)], median 45 days; p = 0.01). However, when interrogated in multivariable analysis, the variable ‘protocol’ no longer remained significant after accounting for age, reflecting the younger mean age of dogs treated with CHOP than ‘Other’ protocols (93 versus 123 months; p = 0.05). (C) Dogs receiving rescue therapy had longer OS (median 322 days) than those not receiving rescue therapy (174 days, median ratio 0.54; p = 0.049). (TIF) [file pone.0105027.s002.tif]
