# Supplementary material for: Dissecting the Regulatory Microenvironment of a Large Animal Model of Non-Hodgkin Lymphoma: Evidence of a Negative Prognostic Impact of FOXP3+ T Cells in Canine B Cell Lymphoma
Source: PLoS One. 2014 Aug 13;9(8):e105027. doi: 10.1371/journal.pone.0105027 (PMC4132014; doi:10.1371/journal.pone.0105027)
Supplement: Table S1 — Cytomorphological criteria for the assessment of lymphoma cases. (DOC) [file pone.0105027.s003.doc]

**Table S1: Cytomorphological criteria for the assessment of lymphoma cases**

| **Feature** | **Criterion / descriptor** |
| --- | --- |
| Nucleus | Size |
|  | Placement |
|  | Shape |
|  | Chromatin pattern |
| Nucleoli | Number |
|  | Size |
|  | Prominence |
|  | Placement |
| Cytoplasm | Amount |
|  | Color |
|  | Presence of Golgi and vacuoles |
| Mitoses (40-50x) | Frequency |
| Macrophages (tingible body) | Frequency |
| Grade | High/low |
