# Supplementary material for: Dissecting the Regulatory Microenvironment of a Large Animal Model of Non-Hodgkin Lymphoma: Evidence of a Negative Prognostic Impact of FOXP3+ T Cells in Canine B Cell Lymphoma
Source: PLoS One. 2014 Aug 13;9(8):e105027. doi: 10.1371/journal.pone.0105027 (PMC4132014; doi:10.1371/journal.pone.0105027)
Supplement: Table S2 — Signalment, therapy and immunophenotype of B cell lymphoma dogs. Abbreviations: mo, months; m, male; f, female; n, neutered; e, entire; ND, not determined; chemotherapy agents: CHOP, protocol in which cyclophosphamide (C), doxorubicin (H), vincristine (O) and prednisolone (P) are administered; COP, protocol in which cyclophosphamide (C), vincristine (O) and prednisolone (P) are administered; L, lomustine; Cy, cytosine arabinoside; Ap, L-asparaginase; Chl, chlorambucil; VCAA, protocol in which vincristine (V), cyclophosphamide (C), L-asparaginase (A) and doxorubicin (A) are administered; LMP, protocol in which chlorambucil (L), methotrexate (M) and prednisolone (P) are administered; DMAC, protocol in which dexamethasone (D), melphelan (M), actinomycin-D (A) and cytosine arabinoside (C) are administered; Ma, masitinib; Vb, vinblastine; Pr, procarbazine; -, no rescue therapy administered (Rescue therapy) or remission not achieved (TTR); +, no progression (PFS) or alive at conclusion of study (OS) and therefore censored from survival analysis. Notes: The immunophenotype lists the per cent positive staining for the listed antigen; 1: these cases were classified as B cell lymphomas with aberrant CD5 expression. (DOC) [file pone.0105027.s004.doc]

**Table S2: Signalment, therapy and immunophenotype of B cell lymphoma dogs**

| **Breed** | **Age**  **(mo)** | **Sex** | **Neutering**  **status** | **Bodyweight**  **(kg)** | **Body**  **condition** | **Initial**  **therapy** | **Rescue**  **therapy** | **TTR**  **(days)** | **PFS**  **(days)** | **OS**  **(days)** | **Immunophenotype** | | |
| --- | --- | --- | --- | --- | --- | --- | --- | --- | --- | --- | --- | --- | --- |
|  |  |  |  |  |  |  |  |  |  |  | **CD21** | **CD79b** | **CD5** |
| Afghan hound | 60 | m | e | 31 | Optimal | CHOP | - | 21 | 361+ | 361+ | 56.9 | 83.8 | 2.4 |
| Australian silky | 160 | m | n | Not recorded | Under-conditioned | None | - | - | - | - | 50.0 | 66.8 | 1.5 |
| Bernese mountain dog | 77 | f | n | 53 | Over-conditioned | CHOP | COP; L,Cy,Ap | 20 | 250 | 521 | 96.1 | ND | 0.1 |
| Border collie | 116 | m | e | 22 | Optimal | CHOP | P,Ap,L | 49 | 26 | 294 | 82.9 | 88.6 | 5.3 |
| Bullmastiff | 81 | f | e | 49 | Optimal | CHOP | - | 21 | 408 | 458 | 96.4 | 99.0 | 0.7 |
| Cocker spaniel | 64 | m | n | 21 | Over-conditioned | CHOP | - | 6 | 77 | 120+ | 60.0 | 46.3 | 5.3 |
| Doberman pinscher | 33 | f | e | 31 | Optimal | CHOP | - | 22 | 305 | 305 | 57.6 | 60.0 | 4.98 |
| Flat-coated retriever | 108 | m | n | 34 | Over-conditioned | COP | Chl,P | 15 | 117 | 249 | 0.2 | 99.7 | 21.0 |
| German shepherd dog | 118 | m | n | 30 | Under-conditioned | CHOP | Ap,L | 8 | 246 | 322 | 76.8 | ND | 13.2 |
| Golden retriever | 166 | f | n | 26 | Under-conditioned | Other (Cy, A) | - | 2 | 24 | 37 | 79.8 | 96.3 | 1.6 |
| Jack Russell terrier | 112 | m | e | 9 | Optimal | CHOP | Chl,P; Ap,L; Cy | - | 60 | 169 | 1.8 | 92.7 | 4.3 |
| Labrador retriever | 113 | f | n | 23 | Optimal | Other (O,C) | Chl,P | 12 | 34 | 619+ | 68.2 | 52.5 | 64.0^1^ |
| Labrador retriever | 147 | f | e | 34 | Optimal | CHOP | - | 12 | 128 | 135 | 81.8 | 95.5 | 0.8 |
| Shar-pei | 127 | m | n | 23 | Optimal | COP | VCAA; LMP | 32 | 102 | 314+ | 51.8 | 62.7 | 6.6 |
| Shetland sheepdog | 82 | m | e | 9 | Not recorded | CHOP | - | 15 | 174 | 174 | 86.2 | 89.0 | 1.1 |
| Shih tzu | 68 | m | e | 9 | Under-conditioned | CHOP | DMAC; Ap,L,Ma,P | 40 | 62 | 85 | 94.5 | ND | 6.3 |
| Spaniel cross | 115 | f | n | 12 | Over-conditioned | COP | - | 45 | 144 | 174 | 90.6 | 98.0 | 0.2 |
| Springer spaniel | 126 | m | n | 24 | Over-conditioned | CHOP | CHOP; Ap,L | 7 | 176 | 448+ | 82.8 | 90.0 | 0.1 |
| Staffordshire bull terrier | 89 | m | n | 23 | Optimal | Other (A, L, Cy) | - | - | 28 | 102 | 96.6 | ND | 4.9 |
| Tibetan terrier | 171 | m | e | 10 | Optimal | CHOP | - | - | 42 | 42 | 11.8 | 91.9 | 77.0^1^ |
| Undefined mixed breed | 144 | f | e | 22 | Optimal | P | - | - | 45 | 45 | 93.3 | ND | 3.0 |
| West Highland white terrier | 49 | f | n | 8 | Optimal | CHOP | CHOP; Ap, L; DMAC; Vb; Pr | 28 | 254 | 486 | 92.6 | ND | 9.9 |
