# Supplementary material for: Dissecting the Regulatory Microenvironment of a Large Animal Model of Non-Hodgkin Lymphoma: Evidence of a Negative Prognostic Impact of FOXP3+ T Cells in Canine B Cell Lymphoma
Source: PLoS One. 2014 Aug 13;9(8):e105027. doi: 10.1371/journal.pone.0105027 (PMC4132014; doi:10.1371/journal.pone.0105027)
Supplement: Table S3 — Signalment, therapy and immunophenotype of T cell lymphoma dogs. Abbreviations: mo, months; m, male; f, female; n, neutered; e, entire; ND, not determined; chemotherapy agents: see Table S2 and Dex, dexamethasone; -, no rescue therapy administered (Rescue therapy) or remission not achieved (TTR); +, no progression (PFS) or alive at conclusion of study (OS) and therefore censored from survival analysis. Notes: The immunophenotype lists the per cent positive staining for the listed antigen. (DOC) [file pone.0105027.s005.doc]

**Table S3: Signalment, therapy and immunophenotype of T cell lymphoma dogs**

| **Breed** | **Age**  **(mo)** | **Sex** | **Neutering**  **status** | **Bodyweight**  **(kg)** | **Body**  **condition** | **Initial**  **therapy** | **Rescue**  **therapy** | **TTR**  **(days)** | **PFS**  **(days)** | **OS**  **(days)** | **Immunophenotype** | | | | |
| --- | --- | --- | --- | --- | --- | --- | --- | --- | --- | --- | --- | --- | --- | --- | --- |
|  |  |  |  |  |  |  |  |  |  |  | **CD21** | **CD79b** | **CD5** | **CD4** | **CD8** |
| Border collie | 144 | m | e | 26 | Optimal | CHOP | Ap, L; DMAC; LMP | 114 | 160 | 314 | 22.9 | ND | 69.4 | 77.3 | 52.9 |
| Boxer | 71 | m | e | 32 | Optimal | Other (Ap, L, P) | - | - | 45 | 68 | 1.5 | 29.9 | 96.7 | 85.7 | 8.21 |
| Boxer | 79 | f | n | 28 | Optimal | CHOP | Dex | 28 | 210 | 230 | 3.0 | 27.8 | 6.8 | 93.4 | 3.16 |
| Boxer | 111 | f | n | 21 | Optimal | Other (Chl) | - | - | 6 | 6 | 2.6 | 2.1 | 84.3 | 98.6 | 0.59 |
| Boxer | 87 | f | n | 28 | Optimal | None | - | - | - | - | 1.5 | 0.5 | 82.5 | 99.9 | 0.76 |
| Cocker  spaniel | 79 | f | n | 16 | Over-conditioned | CHOP | Ap, L | 21 | 487+ | 490+ | 3.5 | 42.1 | 91.6 | 97.3 | 2.89 |
| Cocker  spaniel | 132 | f | n | 13 | Over-conditioned | COP | Ap, L, Cy | 2 | 17 | 63 | 0.39 | 47.1 | 80.2 | 37.7 | 45.4 |
| Dogue de  Bordeaux | 58 | m | e | 54 | Under-conditioned | Other (Ap, L, P) | H, P, L | 60 | 118 | 163 | 4.6 | ND | 70.2 | 95 | 1.13 |
| English springer spaniel | 163 | f | e | 21 | Optimal | CHOP | C | 26 | 98 | 151 | 11.4 | ND | 9.64 | ND | ND |
| Golden retriever | 100 | m | n | 39 | Over-conditioned | CHOP | - | 2 | 13 | 13 | 1.2 | ND | 78.2 | 91.9 | 1.27 |
| Labrador retriever | 88 | f | n | 35 | Over-conditioned | Other (Ma) | L | - | 45 | 515+ | 0.8 | 40.7 | 96.1 | 7.01 | 37.6 |
| Labrador retriever | 98 | m | n | 30 | Optimal | CHOP | Ap, L, Chl, P | 58 | 172 | 267 | 4.8 | 35.5 | 85.5 | 88.2 | 8.19 |
| Labrador retriever | 145 | m | n | 36 | Optimal | CHOP | Ap, L, Chl, P | - | 63 | 63 | 3.2 | 18.3 | 78.3 | 98.4 | 2.17 |
| Shetland sheepdog | 69 | m | e | 9.5 | Optimal | CHOP | - | - | 3 | 5 | 3.8 | 19.8 | 92.5 | 89.3 | 41.2 |
