# Supplementary material for: Dissecting the Regulatory Microenvironment of a Large Animal Model of Non-Hodgkin Lymphoma: Evidence of a Negative Prognostic Impact of FOXP3+ T Cells in Canine B Cell Lymphoma
Source: PLoS One. 2014 Aug 13;9(8):e105027. doi: 10.1371/journal.pone.0105027 (PMC4132014; doi:10.1371/journal.pone.0105027)
Supplement: Table S4 — Disease subtype and signalment of reactive hyperplasia dogs. Abbreviations: mo, months; m, male; f, female; n, neutered; e, entire. (DOC) [file pone.0105027.s006.doc]

**Table S4: Disease subtype and signalment of reactive hyperplasia dogs**

| **Disease subtype** | **Breed** | **Age (mo)** | **Sex** | **Neutering status** | **Bodyweight (kg)** | **Body condition** |
| --- | --- | --- | --- | --- | --- | --- |
| Dermatitis | Fox terrier | 156 | m | n | 13 | Optimal |
| Immune-mediated | French bull dog | 72 | m | n | 11 | Optimal |
| Dermatitis | Jack Russell terrier | 110 | f | n | 12 | Over-conditioned |
| Distant neoplasia | Labrador retriever | 79 | m | n | 31 | Optimal |
| Dermatitis | Fox terrier | 41 | m | n | 11 | Over-conditioned |
| Systemic infection | Rottweiler | 115 | m | e | 49 | Under-conditioned |
| Immune-mediated | Cocker spaniel | 104 | m | n | 15 | Optimal |
| Immune-mediated | Border collie | 114 | f | n | 17 | Under-conditioned |
| Distant neoplasia | Labrador retriever cross | 105 | f | n | 25 | Optimal |
| Dermatitis | Labrador retriever | 144 | f | e | 34 | Optimal |
| Distant neoplasia | German shepherd dog | 92 | f | n | 36 | Optimal |
| Distant neoplasia | Patterdale terrier | 112 | f | n | 11 | Optimal |
| Distant neoplasia | Mixed breed | 136 | f | n | 24 | Optimal |
| Immune-mediated | Irish terrier | 15 | m | n | 15 | Optimal |
