# Supplementary material for: Dissecting the Regulatory Microenvironment of a Large Animal Model of Non-Hodgkin Lymphoma: Evidence of a Negative Prognostic Impact of FOXP3+ T Cells in Canine B Cell Lymphoma
Source: PLoS One. 2014 Aug 13;9(8):e105027. doi: 10.1371/journal.pone.0105027 (PMC4132014; doi:10.1371/journal.pone.0105027)
Supplement: Table S5 — Signalment of mast cell tumor dogs. Abbreviations: mo, months; f, female; n, neutered; e, entire. (DOC) [file pone.0105027.s007.doc]

**Table S5: Signalment of mast cell tumor dogs**

| **Breed** | **Age (mo)** | **Sex** | **Neutering status** | **Bodyweight (kg)** | **Body condition** |
| --- | --- | --- | --- | --- | --- |
| Golden retriever | 129 | f | n | 39 | Over-conditioned |
| Golden retriever | 176 | f | n | 33 | Optimal |
| Labrador retriever | 147 | f | e | 34 | Optimal |
| Rottweiler | 102 | f | n | 43 | Over-conditioned |
| Undefined mixed breed | 84 | f | n | 24 | Optimal |
| Labrador retriever | 147 | f | n | 30 | Over-conditioned |
